# Nutrient-free biorefinery of corn steep water into lactic acid by *Bacillus licheniformis* OP16-2 under thermo-alkaline conditions with a pilot-scale assessment

**DOI:** 10.1038/s41598-026-35828-4

**Published:** 2026-02-02

**Authors:** Mohamed T. Selim, Salem S Salem, Ehab F. El-Belely, Amr Fouda, Mohamed Ali Abdel-Rahman

**Affiliations:** Department of Botany and Microbiology, Faculty of Science, Al-Azhar University, Nasr City, Cairo, 11884 Egypt

**Keywords:** *Bacillus licheniformis*, LA production, Corn steep water, Fed-batch fermentation, Biochemistry, Biological techniques, Biotechnology, Environmental sciences, Microbiology

## Abstract

Lactic acid (LA) is utilized across multiple industries, including polymers, chemicals, cosmetics, and food. Its production from lignocellulosic biomass offers a promising solution to overcome challenges in the production process, such as reducing costs and enhancing environmental sustainability, while also increasing the value of biomass. However, the required pretreatment of lignocellulosic materials to release fermentable sugars generates inhibitory compounds that affect microbial fermentation, alongside the potential risk of contamination by mesophilic and neutrophilic microorganisms. In this study, a strain of *B. licheniformis* was isolated, selected, and identified as a lactic acid producer utilizing corn steep water (CSW) as the sole source of carbon and nitrogen for LA production. This selection was based on the strain’s tolerance to high temperatures and inhibitory compounds, including sodium metabisulfite, sodium chloride, sodium acetate, and formic acid. Sequential optimization of substrate, culture medium, and fermentation parameters was performed using both classical and advanced statistical techniques, without the need for additional nutrient supplementation. Thermo-Alkaline lactic acid production with a pilot-scale assessment was evaluated. Using multi-pulse fed-batch fermentation in a 50 L bioreactor, the system was operated at 45 °C with pH controlled at 8.49 ± 0.30, achieved LA concentration at 152.6 ± 1.15 g/L with a high yield of 0.93 ± 0.02 g/g, and a total productivity of 0.940 ± 0.005 g/L/h after 162 h., starting with an initial CSW concentration of 80 g/L. To our knowledge, this represents the first report of *B. licheniformis* being utilized for LA production from untreated CSW as a low-cost substrate, without any additional treatments or supplements.

## Introduction

LA is a valuable product with broad industrial applications, including its use in the food and beverage industries, pharmaceuticals, cosmetics, leather processing, and textile manufacturing^1–3^. In the food and associated industries, it is a common preservative, natural addition, solvent, curing agent, flavoring ingredient, and buffering agent. Both chemical and biotechnological fermentation procedures can produce LA. While the chemical procedures only generate a racemic mixture of dl-LA, the latter strategy is preferred since it can produce pure LA forms and uses renewable substrates^1,4–6^. The global production of LA is predominantly achieved through microbial fermentation processes^7–9^. In 2021, the global LA market was valued at approximately USD 2.9 billion and is projected to expand at a compound annual growth rate (CAGR) of 8.0% between 2022 and 2030 (https://www.grandviewresearch.com/(.

The high cost of conventional carbon sources renders large-scale, fermentation-based LA production economically unfeasible at present^10,11^. Consequently, there is an urgent need for researchers to explore cost-effective and renewable alternative carbon sources. Initial research efforts primarily focused on food-grade substrates rich in readily fermentable sugars^12^. Concerns over food security have raised ethical and practical issues regarding the use of edible feedstocks. As a solution, residual biomass from agricultural and forestry sources has been explored, demonstrating effectiveness in producing high yields of LA^13–16^. The industrialization of LA production from lignocellulosic biomass is still limited by its high lignin content, even after addressing concerns about food security. Delignification requires energy-intensive and complex pretreatment processes, which significantly elevate production costs^17,18^. Therefore, corn steep water (CSW) was investigated as a cost-effective and promising alternative carbon source to overcome these limitations^19^. One of the main byproducts produced during the wet-milling of corn is CSW^20,21^. CSW serving not only as a carbon source but also as a nitrogen source for microbial fermentation. It has been added to LA fermentation operations as a nitrogen supply supplement^9,22,23^, as an enzyme supplement^24^, and for ethanol production^25^ at low concentrations. Due to its high nutritional content, fermentative media provide a conducive environment for microbial growth, which increases the risk of contamination by mesophilic and neutral LA bacteria and creates an additional challenge to achieving efficient LA fermentation^18,26^. Therefore, employing strains that are tolerant to alkaline conditions and high temperatures would significantly reduce the risk of contamination and promote non-sterile (open) fermentation, thereby decreasing energy consumption during the production process^27^.

In comparison to lactic acid bacteria, *Bacillus* strains offer several advantages for reducing lactic acid production costs, including (1) their ability to ferment substrates at elevated temperatures^28,29^, (2) they are capable of growing in low-cost media, such as mineral media, and can utilize inexpensive nitrogen sources like corn steep liquor or ammonium sulfate ((NH₄)₂SO₄)^30,31^; (3) they are capable of utilizing various sugars present in lignocellulosic biomass^32,33^; (4) they are capable of utilizing xylose and producing LA through homo-fermentation *via* pentose phosphate pathway, converting three molecules of xylose into five molecules of LA^34^; and (4) many species are alkaliphilic, which helps to reduce the risk of contamination during the fermentation process^35^.

Furthermore, optimization is essential for reducing production time and costs, particularly in large-scale manufacturing^36^. The traditional ‘one-factor-at-a-time’ optimization method overlooks the interactions between all parameters, changing one variable while holding the others constant, resulting in time-consuming and expensive processes. A more cost-effective, time-efficient, and efficient way to optimize biochemical and biotechnological processes, on the other hand, is through statistical techniques like response surface methodology (RSM)^37,38^. A factorial experimental design was employed to assess the effects of various variables, involving the modification of all parameters between experiments^39^. The impact of experimental components and their interactions is frequently studied using factorial designs, particularly to determine how the influence of one factor changes in response to different levels of other factors^40^.

Most microbial LA fermentations are performed in batch mode; however, this approach has several disadvantages, including substrate and product inhibition, as well as long fermentation times that can reduce both the efficiency and overall productivity of LA production^8,26,41^. In contrast, the fed-batch fermentation technique is primarily employed to increase LA concentration and mitigate substrate inhibition^18,42^. Substrate concentrations exceeding the critical level can inhibit microbial strains, induce cell lysis, ultimately reducing both sugar consumption and LA production^43^. To mitigate this challenge, fed-batch fermentation was implemented by maintaining the substrate concentration at an optimal level and periodically adding nutrients to the fermentation broth^44^ without removing any components from the fermentation process^43^.

This study aimed to determine whether stress-tolerant LA producers could produce LA utilizing CSW as their sole carbon and nitrogen sources. Stress-tolerant bacterial isolates were successfully screened and identified to select the most effective LA-producing strain. In addition, we sequentially optimized the substrate, culture medium, and fermentation conditions using both classical and modern statistical techniques to enhance the bioconversion of CSW to LA. We also evaluated the feasibility of CSW fermentation without additional nutrient supplementation. Ultimately, the study evaluated cost-effective and sustainable long-term LA production by batch and multi-pulse fed-batch fermentation at a pilot scale (50 L fermentor).

## Materials and methods

### CSW collection and fermentative media

CSW was obtained from the outflow of a steeping tank at a commercial wet-milling facility for maize in the El-Sharqia Governorate of Egypt. The sample materials were stored at -20 °C to preserve them for further examination. A modified yeast extract dextrose (MYD) was utilized for bacterial screening and isolation, containing the following (g/L): agar, 15; CSW, 20 as the sole carbon source; and yeast extract (YE), 5. A 5 N NaOH solution was used to adjust the pH at 9.0. One mL of bacterial culture from the glycerol stock was inoculated at 9.0 mL of MYD medium to prepare a refreshment culture for the fermentation experiment, which was then incubated for 24 h at 50 °C. After preparing the preculture, 1.0 mL of the refreshed culture was transferred into 9.0 mL of the same fermentative medium in a test tube and incubated at 50 °C for 24 h. At a 10% (*v/v*) concentration, this culture was subsequently employed as the inoculum for the primary fermentation cultures. Different concentrations of CSW sugar were added to the fermentative media as described in each experiment.

### Isolation and screening of LA producers

An Erlenmeyer flask (125 mL) contain 40.0 mL of MYD medium (pH 9.0) supplemented with 20.0 g/L of CSW, was inoculated with one gram of soil samples obtained from different governorates in Egypt. The flasks were incubated at 50 °C for 48 h. After that, a 100 µL sample from each flask was spread onto MYD agar and incubated for 24 h., at 50 °C. Visible colonies of bacteria were re-spread on the MYD agar for purification. Acid-producing bacterial isolates were selected after the pure isolates were cultivated on MYD agar supplemented with 5.0 g/L CaCO₃. Bacterial isolates were kept at − 80 °C in 30% glycerol. Broth media with 20 g/L of CSW-sugar was used for additional screening, and the cultures were incubated for 48 h at 50, 55, and 60 °C. Different concentrations of sodium metabisulfite (1.0–8.0 g/L), sodium chloride (2.5–10%), sodium acetate (5–20 g/L), and formic acid (2.5–10 g/L) were added separately to MYD with CSW as the only carbon source to evaluate the effects of inhibitors on the sugar consumption, LA concentration and yield.

### Characterization and identification of the most potent isolate

The most potent lactic acid producer, designated OP16-2 which was reported in our previous study^19^, was subjected to molecular identification by extracting genomic DNA using a modified method as described previously^45,46^. The 16 S rRNA gene sequence of the isolated strain was added to GenBank with accession number ON650717.

### Optimization of fermentation conditions using OFAT

Experiments were conducted to optimize fermentation conditions using the “one-factor-at-a-time” (OFAT) method to study the effects of various factors on LA production by *B. licheniformis* OP16-2. First, the impact of sugar concentrations (20.0, 40.0, 60.0, 80.0, 100 g/L) from CSW on LA production was tested in 50 mL fermentation flasks, with the initial pH adjusted to 9.0 and incubation at 50 °C for 48 h. The effect of different temperatures (35–55 °C) on production was also studied at the optimal sugar concentration, with the pH maintained at 9.0. Additionally, the effect of inoculum size (ranging from 2.5 to 12.5%, *v/v*) was evaluated in MYD at 45 °C for 48 h. The effect of different neutralizing agents (NaOH and CaCO₃) for controlling pH during fermentation was also examined, with NaOH 5 N added or CaCO₃ used at a concentration of 0.5 g/g carbon source. The influence of varying concentrations of YE (0.0–5.0 g/L) on LA production was investigated in MYD at 45.0 °C for 96 h. Finally, the effect of different initial pH values (8.0, 8.5, 9.0, and 9.5) on LA production was investigated under all the previously optimized conditions.

### Optimization by RSM in batch fermentation

With the central composite design (CCD) being the most widely used and successful optimization approach, statistical optimization was carried out utilizing RSM. A 2-level, 5-factor (2^5^) complete factorial-CCD was used to assess the effects of temp., pH, inoculum size, YE (with and without addition), and sugar content. Table 1 presents the CCD matrix, along with the actual experimental run data for each factor at five levels. The response variables from the CCD were fitted using a full-quadratic multiple regression model, considering the four continuous factors (sugar concentration, temperature, pH, and inoculum size) and the categorical factor (YE). Analysis of variance (ANOVA) was performed to assess the statistical significance of the linear, quadratic, and interaction terms.

**Table 1:** Optimization of 2^5^ full factorial CCD based on all combinations of low and high levels of interacting factors.

Design points		Factors					Responses			
Run Order	Point Type	Sugar Conc. (g/L)	Temp. (°C)	Inocula Size (%, *v/v*)	pH	YE (5 g/L)	Consumed sugar (g/L)	Lactic acid (g/L)	Lactic acid yield (g/g)	Total productivity (g/L/h)
1	1	50	40	10.0	8.0	with	28.5	25.3	0.89	0.26
2	1	110	40	10.0	8.0	with	20.4	17.7	0.87	0.19
3	1	50	50	10.0	8.0	with	28.9	25.0	0.86	0.26
4	1	110	50	10.0	8.0	with	28.3	24.8	0.88	0.26
5	1	50	40	15.0	8.0	with	38.8	35.3	0.91	0.37
6	1	110	40	15.0	8.0	with	49.5	43.3	0.87	0.45
7	1	50	50	15.0	8.0	with	38.3	34.9	0.91	0.36
8	1	110	50	15.0	8.0	with	48.5	42.2	0.87	0.44
9	1	50	40	10.0	9.0	with	28.2	25.1	0.89	0.26
10	1	110	40	10.0	9.0	with	20.2	17.9	0.89	0.19
11	1	50	50	10.0	9.0	with	28.8	25.0	0.87	0.26
12	1	110	50	10.0	9.0	with	28.2	24.9	0.89	0.26
13	1	50	40	15.0	9.0	with	38.9	35.2	0.91	0.37
14	1	110	40	15.0	9.0	with	49.6	43.2	0.87	0.45
15	1	50	50	15.0	9.0	with	38.2	34.8	0.91	0.36
16	1	110	50	15.0	9.0	with	48.6	42.3	0.87	0.44
17	-1	20	45	12.5	8.5	with	18.3	16.5	0.90	0.17
18	-1	140	45	12.5	8.5	with	28.3	24.1	0.85	0.25
19	-1	80	35	12.5	8.5	with	39.5	33.2	0.84	0.35
20	-1	80	55	12.5	8.5	with	49.2	43.5	0.88	0.45
21	-1	80	45	7.5	8.5	with	35.6	31.2	0.88	0.33
22	-1	80	45	17.5	8.5	with	43.6	39.3	0.90	0.41
23	-1	80	45	12.5	7.5	with	28.9	25.3	0.88	0.26
24	-1	80	45	12.5	9.5	with	25.9	22.3	0.86	0.23
25	0	80	45	12.5	8.5	with	79.9	75.6	0.95	0.79
26	0	80	45	12.5	8.5	with	79.8	75.7	0.95	0.79
27	0	80	45	12.5	8.5	with	79.7	75.2	0.94	0.78
28	0	80	45	12.5	8.5	with	79.5	74.9	0.94	0.78
29	0	80	45	12.5	8.5	with	79.8	74.9	0.94	0.78
30	0	80	45	12.5	8.5	with	79.9	75.9	0.95	0.79
31	0	80	45	12.5	8.5	with	79.9	75.2	0.94	0.78
32	1	50	40	10.0	8.0	without	27.8	24.7	0.89	0.26
33	1	110	40	10.0	8.0	without	19.7	17.1	0.87	0.18
34	1	50	50	10.0	8.0	without	28.3	24.3	0.86	0.25
35	1	110	50	10.0	8.0	without	27.6	24.2	0.88	0.25
36	1	50	40	15.0	8.0	without	38.1	34.7	0.91	0.36
37	1	110	40	15.0	8.0	without	48.9	42.7	0.87	0.45
38	1	50	50	15.0	8.0	without	37.6	34.3	0.91	0.36
39	1	110	50	15.0	8.0	without	47.9	41.6	0.87	0.43
40	1	50	40	10.0	9.0	without	27.5	24.5	0.89	0.26
41	1	110	40	10.0	9.0	without	19.5	17.3	0.89	0.18
42	1	50	50	10.0	9.0	without	28.2	24.3	0.86	0.25
43	1	110	50	10.0	9.0	without	27.5	24.3	0.88	0.25
44	1	50	40	15.0	9.0	without	38.2	34.6	0.90	0.36
45	1	110	40	15.0	9.0	without	49.0	42.6	0.87	0.44
46	1	50	50	15.0	9.0	without	37.5	34.2	0.91	0.36
47	1	110	50	15.0	9.0	without	48.0	41.7	0.87	0.43
48	-1	20	45	12.5	8.5	without	17.9	16.1	0.90	0.17
49	-1	140	45	12.5	8.5	without	27.9	23.7	0.85	0.25
50	-1	80	35	12.5	8.5	without	39.1	32.8	0.84	0.34
51	-1	80	55	12.5	8.5	without	48.8	43.1	0.88	0.45
52	-1	80	45	7.5	8.5	without	35.2	30.8	0.87	0.32
53	-1	80	45	17.5	8.5	without	43.2	38.9	0.90	0.41
54	-1	80	45	12.5	7.5	without	28.5	24.9	0.87	0.26
55	-1	80	45	12.5	9.5	without	25.5	21.9	0.86	0.23
56	0	80	45	12.5	8.5	without	78.7	74.5	0.95	0.78
57	0	80	45	12.5	8.5	without	78.6	74.6	0.95	0.78
58	0	80	45	12.5	8.5	without	78.6	75.1	0.95	0.78
59	0	80	45	12.5	8.5	without	78.5	74.5	0.95	0.78
60	0	80	45	12.5	8.5	without	78.2	73.9	0.94	0.77
61	0	80	45	12.5	8.5	without	78.5	74.2	0.95	0.77
62	0	80	45	12.5	8.5	without	79.0	74.5	0.94	0.78

#### Experiments setup

To assess the effect of CSW concentration on LA production by the OP16-2 strain, a total of 62 flasks were used, with 31 containing 5 g/L of yeast extract and 31 without YE supplementation. The experimental conditions included five levels of sugar concentration (20–100 g/L), five temperature levels (35–55 °C), five inoculum sizes (5–15%), and five pH levels (7.5–9.5). During fermentation, 5 N NaOH was added as a neutralizing agent to maintain the pH, and the fermentation process lasted for 84 h.

#### Optimization curves

Optimization curves for determining the ideal combination of interacting parameters that enhance LA productivity and yield were created using the response optimizer, a component of the statistics program Minitab^®^-DoE (Design of Experiments). On a scale of 0 to 1, individual (d) and composite (D) desirability indices were used to assess how well the anticipated settings maximized the response. Additional confirmation tests (*n* = 10 replicated runs) were conducted at the end of this phase to verify the parameters expected based on the optimization curves.

### Improved fermentation strategies in a bioreactor (50 L) for enhancing LA production

#### Bacterial strain and culture conditions (in pilot-scale)

The stock cultures’ cells were refreshed by growing them in de Man, Rogosa, and Sharpe (MRS) medium, which contained 20.0 g/L glucose, 10.0 g/L peptone, 10.0 g/L beef-extract, 5 g/L yeast-extract (YE), 5 g/L sodium-acetate trihydrate, 2 g/L K_2_HPO_4_, 2 g/L tri-ammonium citrate, 0.1 g/L magnesium sulphate heptahydrate, 0.05 g/L manganese sulphate tetrahydrate, and 1 mL Tween 80. To create the seed culture, 1 mL of the refreshed culture was inoculated into 250 mL of sterile media in a 500 mL conical flask containing the same ingredients. The mixture was then incubated for 18 h. at 45 °C. After 18 h., the seed culture was transferred to 2350 mL of sterile medium (main culture) in a 5000 mL conical flask, containing the same ingredients, and incubated at 45 °C for another 18 h.

#### Batch fermentation in a bioreactor

The preparation of CSW in a 50 L bioreactor was carried out as follows: The CSW was diluted with 17.4 L of distilled water to achieve a total sugar concentration of 80 g/L. The pH was adjusted to 8.49, and the mixture was sterilized in the bioreactor at 121 °C for 15 min. After sterilization, the main culture was inoculated at a concentration of 13.2% (*v/v*) into the bioreactor under sterile conditions, resulting in a total volume of 20 L. The bioreactor temperature was maintained at 45 °C, and the pH was controlled at 8.49 ± 0.30 using an automatic pH control using 10 N NaOH as neutralizing agent for 84 h. Samples were taken every 12 h. to measure all fermentation parameters.

#### Multi-pulse fed-batch fermentation for enhancing LA production (in a bioreactor 50 L)

The multi-pulse-fed batch fermentations were conducted in a 50 L bioreactor under aseptic conditions, with an initial CSW sugar concentration of 80.0 g/L. The bacterial strain, culture conditions, and bioreactor preparation followed the same procedures as those used in batch fermentation. Two sterile feedings, each containing 40 g/L of concentrated CSW sugar, were added at different intervals when the residual sugar concentration reached approximately 40 g/L. A single sterile feeding containing 20 g/L of concentrated CSW sugars was added as the final supplement. The pH was maintained at 8.49 ± 0.30 using an automatic pH control system using 10 N NaOH throughout the fermentation process. Samples were collected at various times to measure all fermentation parameters.

### Analytical and statistical analysis

In our previous study^19^, CSW was analyzed for various properties, including physicochemical properties, presence of inorganic ions, amino acid content, fat- and water-soluble vitamins, and non-protein nitrogenous substances. It also examined the presence of inorganic ions, amino acid content, fat- and water-soluble vitamins, and non-protein nitrogenous substances. Using glucose as the standard, the phenol-sulfuric acid method was employed to measure the amount of consumed sugars during the fermentation studies^47^. The total viable count approach was used to assess bacterial cell growth. The Barker and Summerson technique^48^ was used to measure the LA content in intermittent supernatant samples obtained following ten minutes of centrifugation at 6,000 *rpm*. The LA-productivity (PLA, g/L/h) was calculated by dividing the concentration of LA produced by the fermentation period, whilst LA yield (g/g) was computed as the ratio of LA (g/L) to CSW sugar (g/L). The difference in LA concentrations between two samples was divided by the time interval to get the maximal LA productivity (g/L/h).

Minitab^®^ version 18 (2017) was used to produce and analyze the experimental data for the RSM models. Graphical and statistical software tools supplemented it. The coefficient of determination (R²) obtained from the analysis of variance (ANOVA) was used to assess the model’s efficacy. ANOVA was used to evaluate the data, which were then displayed as mean ± SD. The Fisher test was then used to compare the means at a 0.05 probability level. Sigma Plot v14.0 SPW was used to create surface plots.

## Results and discussion

### Isolation and screening of the most potent lactic acid producers

LA producers were separated from soil samples using CSW-based media in thermo-alkaline conditions (50 °C and pH 9.0) to address issues related to LA fermentation, such as substrate cost and contamination hazards^26^. Ten of the 50 bacterial isolates exhibited high LA production yields (more than 0.81 g/g). A second screening was conducted to assess the LA production capacity of these isolates at higher temperatures of 55 °C and 60 °C. Among the 10 isolates, only three isolates were able to withstand 60° C and produce more than 10.0 g/L of LA (Table 2). Two isolates, WH11-3 and OP16-2, were selected as the potent producers and were chosen for further investigation into the inhibitory compounds present in effluent substrates.

**Table 2:** Effect of temperature variations on the ten most potent isolates’ sugar consumption, LA concentration, and LA yield.

NO.	Isolate code	Catalase Activity	Cell shape	Temperature at 50 °C				Temperature at 55 °C				Temperature at 60 °C			
				pH	C. SU (g/L)^a^	LA conc. (g/L)^b^	Y_LA_ (g/g)^c^	pH	C.SU (g/L)	LA conc (g/L)	Y_LA_ (g/g)^c^	pH	C.SU (g/L)	LA conc (g/L)	Y_LA_ (g/g)^c^
**1**	WH 1–6	Positive	Rod	6.07	18.0	14.6	0.81	6.84	17.4	13.9	0.80	6.76	15.24	9.80	0.64
**2**	SSD 18 − 1	Positive	Rod	4.90	17.7	14.4	0.81	5.06	16.9	10.5	0.62	5.52	15.88	8.62	0.54
**3**	WH 40 − 1	Positive	Rod	5.62	14.5	12.7	0.88	5.18	14.3	11.1	0.78	5.24	10.53	6.35	0.60
**4**	OP 5 − 1	Positive	Rod	5.63	16.9	14.1	0.84	5.19	16.7	10.5	0.63	5.88	16.08	8.97	0.56
**5**	OP 2–2	Positive	Rod	5.84	18.2	16.4	0.90	5.15	14.3	12.3	0.87	5.38	15.99	9.50	0.59
**6**	OP 25 − 2	Positive	Rod	4.51	13.8	12.6	0.91	5.11	12.8	9.2	0.72	5.18	11.91	6.35	0.53
**7**	OP 13 − 1	Positive	Rod	6.91	17.6	16.0	0.91	6.53	16.5	12.0	0.73	6.72	15.58	10.1	0.65
**8**	WH 11 − 3	Positive	Rod	6.91	15.1	13.8	0.91	6.66	17.0	14.7	0.86	6.83	14.02	10.5	0.75
**9**	OP 3 − 2	Positive	Rod	7.31	17.6	14.3	0.81	6.53	17.1	13.6	0.80	6.90	15.25	9.21	0.60
**10**	OP 16 − 2	Positive	Rod	5.90	18.1	17.3	0.95	6.00	16.7	13.9	0.83	5.21	12.05	10.4	0.87

^a^ Consumed sugar after 48 h, ^b^Maximum lactic acid concentration after 48 h, ^c^ Lactic acid yield.

### Effect of stress conditions on LA production

This study investigates the effect of various inhibitors on sugar consumption and LA fermentation by the most effective bacterial isolates. Different concentrations of sodium metabisulfate (1–8 g/L), sodium chloride (2.5–10.0%, *w/v*), sodium acetate (5–20.0 g/L), and formic acid (2.5–10.0 g/L) were individually incorporated into the fermentation medium, utilizing CSW as the sole carbon source (Table 3). The two selected isolates demonstrated high LA yields, ranging from 0.60 to 0.88 g/g for WH11-3 and 0.77 to 0.90 g/g for OP16-2, when sodium metabisulfate concentrations ranged from 1.0 to 4.0 g/L. However, LA yields decreased to 0.22 and 0.62 g/g at 8.0 g/L of sodium metabisulfate for WH11-3 and OP16-2, respectively. Additionally, high lactic acid yields of 0.44 to 0.74 g/g for WH11-3 and 0.54 to 0.93 g/g for OP16-2 were obtained with 2.5 to 7.5% sodium chloride, while lower yields (0.33 and 0.49 g/g) were observed at 10% NaCl for both isolates.

**Table 3:** Impact of several inhibitory substances on the most potent isolates’ LA yield, LA-concentration, and sugar consumption.

Bacterial Isolate	Sodium Metabisulfate				Sodium chloride				Sodium acetate				Formic acid			
	Inhibitor Conc. (g/L)	Consumed sugar (g/L)	LA conc. (g/L)^a^	*Y*_LA_ (g/g)^b^	Inhibitor Conc. (%	Consumed sugar (g/L)	LA conc. (g/L)^a^	*Y*_LA_ (g/g)^b^	Inhibitor Conc. (g/L)	Consumed sugar (g/L)	LA conc. (g/L)^a^	*Y*_LA_ (g/g)^b^	Inhibitor Conc. (g/L)	Consumed sugar (g/L)	LA conc. (g/L)^a^	*Y*_LA_ (g/g)^b^
WH 11 − 3	1	15.2	13.4	0.88	2.5	16.3	12.0	0.74	5	13.3	10.6	0.80	2.5	15.8	14.2	0.90
	2	15.7	10.3	0.66	5.0	14.1	8.27	0.58	10	10.8	8.63	0.79	5.0	13.0	10.3	0.79
	4	5.24	3.12	0.60	7.5	4.14	1.82	0.44	15	11.3	6.31	0.56	7.5	10.1	7.11	0.70
	8	2.31	0.51	0.22	10.0	1.87	0.62	0.33	20	5.03	3.74	0.74	10.0	4.32	2.36	0.55
OP 16 − 2	1	17.9	16.2	0.90	2.5	18.1	16.9	0.93	5	17.2	15.9	0.92	2.5	16.6	14.9	0.89
	2	16.7	13.6	0.81	5.0	16.0	14.2	0.89	10	14.7	12.6	0.85	5.0	13.8	11.9	0.86
	4	8.14	6.28	0.77	7.5	7.94	4.28	0.54	15	6.49	3.77	0.58	7.5	8.69	6.89	0.79
	8	5.61	3.47	0.62	10.0	4.99	2.47	0.49	20	3.88	1.98	0.51	10.0	3.98	2.91	0.73

^a^Maximum lactic acid concentration, ^b^ Lactic acid yield.

A similar pattern was observed for isolates WH11-3 and OP16-2, with LA yields ranging from 0.56 to 0.80 g/g for WH11-3 and 0.58 to 0.92 g/g for OP16-2 when sodium acetate concentrations were between 5.0 and 15.0 g/L. However, at 20.0 g/L of CH_3_COONa, the LA yield decreased to 0.74 and 0.51 g/g, respectively. On the other hand, 2.5 to 7.5 g/L of formic acid produced LA yields of 0.70 to 0.90 g/g for WH11-3 and 0.79 to 0.89 g/g for OP16-2. For both isolates, LA yields of 0.55 and 0.73 g/g were obtained with 10 g/L of formic acid.

Corn is immersed in water containing 0.2% sulfur dioxide, which is generated when sodium metabisulfite breaks down the disulfide bonds in the protein matrix surrounding the starch granules. This process frees the starch granules, enhancing the overall recovery of starch after the protein matrix in the endosperm has been disrupted^49^. To inhibit microbial growth during the steeping process, sulfur dioxide and elevated temperatures are applied in large-scale steeping tanks^50^. Given that the selected strain, OP16-2, showed remarkable stability across various sodium metabisulfate concentrations, we hypothesize that it will be highly effective for LA fermentation. Moreover, the impact of salt stress on growth, survival, and central carbon metabolism hampers the efficiency of LA production^51^. Previous studies have shown that salt stress alters the fatty acid composition of cell membranes in various bacteria^52–54^. Based on our results, isolate OP16-2 demonstrated enhanced stability under high-stress or inhibitory compounds that may be present in the waste materials being studied. As a result, it was chosen for further characterization and analysis.

**Fig. 1:**
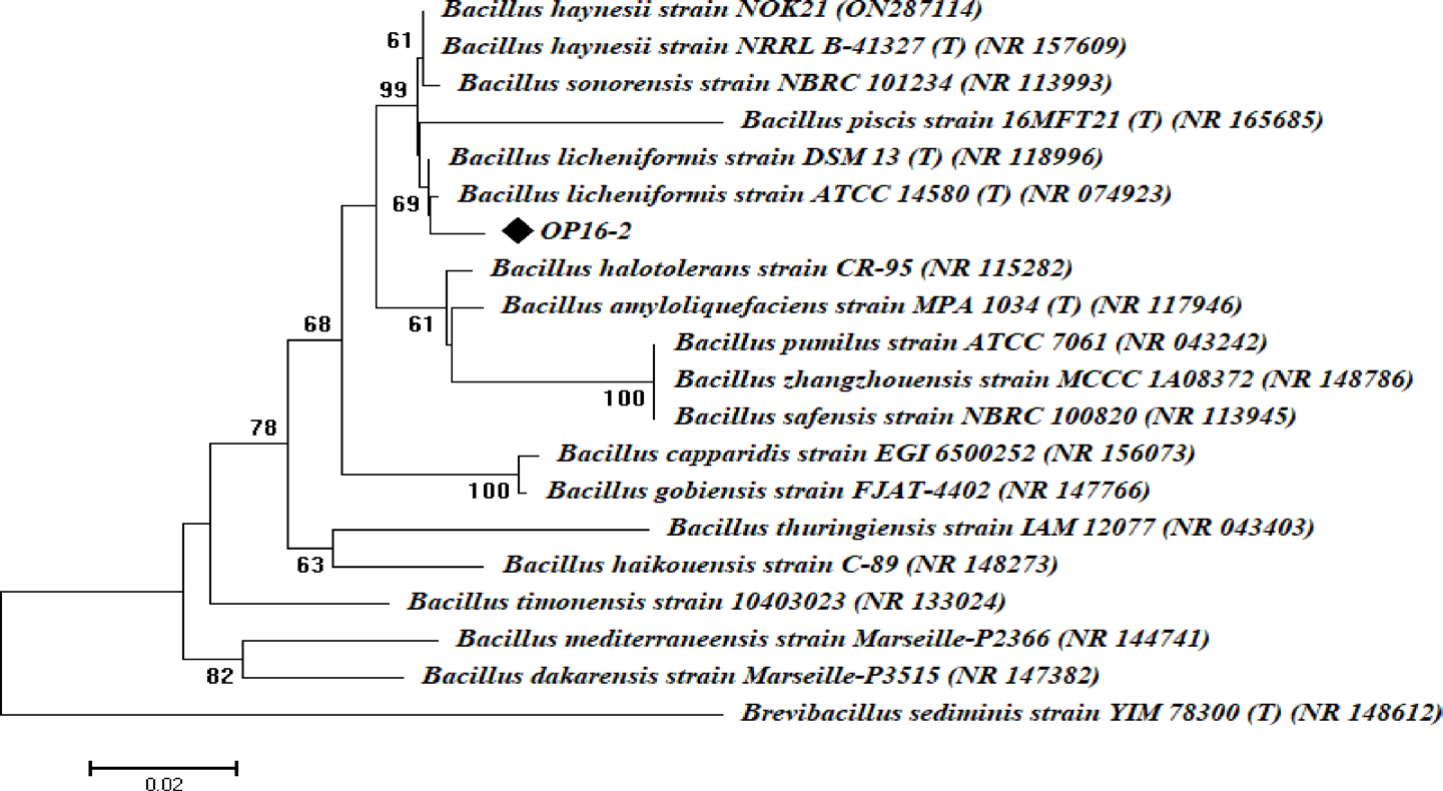
Phylogenetic analysis of the 16S rRNA sequences of the isolate was performed by comparing them with those in the NCBI. The symbol ♦ represents the 16S rRNA gene fragments.

### Characterization of OP16-2 isolate

Morphological and growth characters were obtained as indicated in Table 4 and showed that the OP16-2 isolate is short rods and has a white colony on the plate. It can grow at a wide temperature and pH ranges and can ferment several sugars including glucose, fructose, sucrose, maltose, lactose, cellulose and starch. This isolate was molecularly identified as *Bacillus licheniformis* OP16-2 through 16S rRNA sequencing **(**Fig. 1**).**

**Table 4:** Characterization of *OP16-2 isolate.*

Character	OP16-2
*Cell morphology*	Rod-shaped
*Colony Color*	White
*Colony shape*	Convex
*Gram stain*	+
*Catalase activity*	+
*Fermentation type*	Homo
*Growth temperature (20–60 °C)*	+
*Growth pH (4.0–11.0)*	+
*Sugar fermentations*	
*Glucose*	+
*Fructose*	+
*Sucrose*	+
*Maltose*	+
*Lactose*	+
*Cellulose*	+
*Starch*	+

+, Positive reaction; -, Negative reaction.

### “One-factor-at-a-time-[OFAT]” optimization

#### Effect of sugar concentrations

Table 5 provides a summary of the profiles and fermentation parameters for LA production at varying sugar concentrations. Cell growth in term of total viable count was increased from 75.3 ± 4.5 × 10¹⁰ CFU/mL at 20.0 g/L to 136.3 ± 4.1 × 10¹⁰ CFU/mL at 80 g/L of total sugars, while decreased to 30.3 ± 1.2 × 10¹⁰ CFU/mL at 100 g/L. Similarly, sugar consumption by *B. licheniformis* OP16-2 was increased from 19.4 ± 0.43 g/L at 20.0 g/L to 57.8 ± 1.05 g/L at 80 g/L, while it decreased to 22.0 ± 1.01 g/L at 100 g/L. LA production followed a similar trend, with final LA concentration increasing from 17.4 ± 0.5 g/L at 20.0 g/L to the highest value of 52.2 ± 1.6 g/L at 80 g/L, then declining to 18.8 ± 1.03 g/L at 100 g/L. LA yield ranged from 0.89 ± 0.1 to 0.90 ± 0.01  g/g of sugar consumed at 20 to 80 g/L, and slightly decreased to 0.85 ± 0.03 g/g at 100 g/L. LA-productivity also increased from 0.36 ± 0.01 g/L/h at 20.0 g/L to 1.0 ± 0.03 g/L/h at 80 g/L, then decreased to 0.39 ± 0.02 g/L/h at 100 g/L. The Maximum LA-productivity ranged from 0.58 ± 0.02 to 1.49 ± 0.06 g/L/h, with the highest value achieved at 80 g/L of total sugars. Overall, comparable LA yield and productivity were observed at sugar concentrations of 20–80 g/L, with the highest LA concentration (52.2 ± 1.6 g/L) and maximum productivity (1.49 ± 0.06 g/L/h) achieved at 80 g/L of total sugars.

**Table 5:** The effect of various CSW concentrations on all fermentation variables.

CSW conc. (g/L)	Total Viable Count (×10^10^) CFU/mL	Consumed Sugar (g/L)	LA conc. (g/L )^a^	Y_LA_ (g/g)^b^	*P*_LA_ (g/L/h)^c^	Max *P*_LA_ (g/L/h)^d^ at the indicated time
20	75.3 ± 4.5 d	19.4 ± 0.43 d	17.4 ± 0.5 d	0.89 ± 0.1 ab	0.36 ± 0.01 d	0.73 ± 0.03 (36)
40	108 ± 3.6 c	35.9 ± 1.05 c	33.1 ± 0.95 c	0.92 ± 0.0 a	0.69 ± 0.01 c	0.80 ± 0.03 (12)
60	125 ± 5 b	49.3 ± 1.1 b	44.3 ± 1.3 b	0.89 ± 0.0 ab	0.92 ± 0.02 b	1.31 ± 0.04 (12)
80	136.3 ± 4.1 a	57.8 ± 1.05 a	52.2 ± 1.6 a	0.90 ± 0.01 ab	1.0 ± 0.03 a	1.49 ± 0.06 (12)
100	30.3 ± 1.2 e	22.0 ± 1.01 d	18.8 ± 1.03 d	0.85 ± 0.03 b	0.39 ± 0.02 d	0.58 ± 0.02 (12)

^a^ Maximum LA concentration after 48 h, ^b^ LA yield, ^c^ LA productivity at the end of fermentation time, ^d^ Maximum LA productivity at the indicated time. Mean ± SD is used to represent the data (*n* = 3). While values in the same column with the same letter are not significantly different, different lower-case letters in the same column are, according to the post-hoc Tukey’s test, significantly different at *p* < 0.05.

With an increase in initial sugar concentrations up to a certain point, several researchers also noted a higher LA concentration^55–57^. The extremely high sugar concentrations increased osmotic pressure, which reduced LA productivity^58^. Several LAB can withstand sugar concentrations higher than 60 g/L, including *Lacticaseibacillus paracasei* subsp. *paracasei* CHB2121^59^, *Enterococcus mundtii* QU 25^8^, *Enterococcus faecalis* CBRD01^60^, and *Lactobacillus mutant* G-03^61^.

#### Effect of temperature

**Fig. 2:**
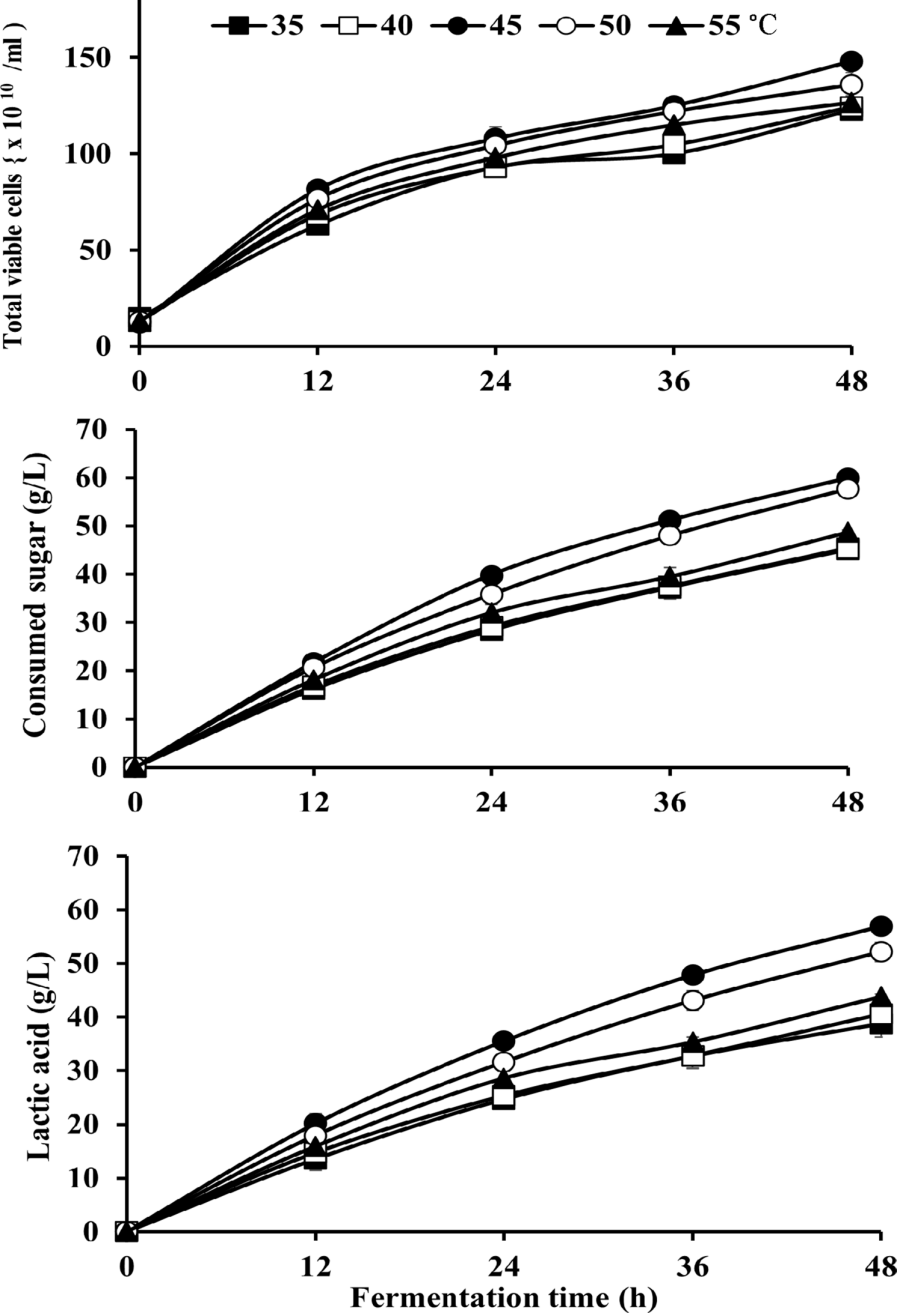
Effect of different temperatures (35–55 °C) on LA production by *Bacillus licheniformis* OP16-2. The standard deviation is less than the size of the symbols if no error bars are seen.

The operating fermentation temperature affects the rate of growth, biochemical reactions, enzyme activities, as well as the substrate-consumption rate and production efficiency of LA^18,56^. The data presented in Fig. 2 shows that the highest cell growth was increased from 122.6 ± 2.0 × 10^10^ CFU/mL at 35 °C to 148 ± 2.0 × 10^10^ CFU/mL at 45 °C, while decreased to 126.6 ± 5.6 × 10^10^ CFU/mL at 55 °C. Similarly, sugar consumption by *B. licheniformis* OP16-2 was increased from 45.1 ± 1.9 g/L at 35 °C to 59.9 ± 1.0 g/L at 45 °C, and then slightly decreased to 48.6 ± 0.5 g/L at 55 °C. A similar trend was observed for LA-production, with the final LA- concentration increasing from 38.7 ± 2.4 g/L at 35 °C to the highest value of 56.9 ± 0.8 g/L at 45 °C, before dropping to 43.7 ± 0.5 g/L at 55 °C. LA productivity also followed this pattern, rising from 0.80 ± 0.05 g/L/h at 35 °C to 1.18 ± 0.01 g/L/h at 45 °C, and then decreasing to the lowest value of 0.89 g/L/h at 55 °C. The highest LA production was observed at 45 °C, indicating that this temperature is optimal for LA production by *B. licheniformis* OP16-2.

#### Effect of inoculum size

To evaluate the effect of varying inoculum sizes on LA-production by *B. licheniformis* OP16-2, fermentation experiments were conducted using inoculum doses ranging from 2.5% to 12.5% (*v/v*) in MYD medium at 45 °C for 48 h. As shown in Table 6, the highest cell growth in term of total viable count was increased from 125.6 ± 4.0 × 10¹⁰ CFU/mL at a 2.5% inoculum to 151.6 ± 1.5 × 10¹⁰ CFU/mL at 12.5%. Similarly, sugar consumption increased from 51.9 ± 2.5 g/L at a 2.5% inoculum to 61.8 ± 1.5 g/L at a 12.5% inoculum. Correspondingly, the final LA concentration increased from 47.0 ± 0.2 g/L to a maximum of 57.4 ± 1.3 g/L as the inoculum size increased from 2.5% to 12.5% (*v/v*).

**Table 6:** Effect of inoculum size of CSW on the growth, sugar consumption, LA concentration, LA yield, LA productivity, and maximum LA productivity by *Bacillus licheniformis* OP16-2.

Inoculum size (%)	Total Viable Count (×10^10^) CFU/mL	Consumed Sugar (g/L )	LA conc. (g/L )^a^	Y_LA_ (g/g)^b^	*P*_LA_ (g/L/h)^c^	Max *P*_LA_ (g/L/h)^d^ at the indicated time
2.5	125.6 ± 4.0 d	51.9 ± 2.5 c	47.0 ± 0.2 d	0.90 ± 0.04 a	0.98 ± 0.0 d	1.2 ± 0.13 (12)
5	131.0 ± 2.6 cd	54.9 ± 2.6 bc	49.8 ± 1.1 c	0.90 ± 0.03 a	1.03 ± 0.02 c	1.3 ± 0.12 (12)
7.5	138.3 ± 7.2 bc	56.7 ± 2.3 abc	52.9 ± 1.1 b	0.93 ± 0.02 a	1.10 ± 0.02 b	1.5 ± 0.12 (12)
10	148.3 ± 1.5 ab	60.7 ± 1.7 ab	57.0 ± 0.8 a	0.93 ± 0.02 a	1.18 ± 0.01 a	1.6 ± 0.15 (12)
12.5	151.6 ± 1.5 a	61.8 ± 1.5 a	57.4 ± 1.3 a	0.92 ± 0.02 a	1.19 ± 0.02 a	1.7 ± 0.18 (12)

^a^ Maximum lactic acid concentration after 48 h, ^b^ Lactic acid yield, ^c^ Lactic acid productivity at the end of fermentation time, ^d^ Maximum lactic acid productivity at the indicated time. Data represented by Mean ± SD (*n* = 3). Different lower-case letters in the same column are significantly different by post hoc Tukey’s test at *p* < 0.05; values in the same column with the same letter are not significantly different.

LA productivity was increased from 0.98 ± 0.0 g/L/h at an inoculum size of 2.5% (*v/v*) to 1.19 ± 0.02 g/L/h at 12.50% (*v/v*). The maximum productivity values ranged between 1.2 ± 0.13 and 1.7 ± 0.18 g/L/h, with the highest recorded at 12.5%. Based on these results, 12.5% (*v/v*) was identified as the optimal inoculum size for maximizing LA production by *B. licheniformis* OP16-2.

Effective fermentation and a shorter lag phase are strongly influenced by inoculum size^9^. Previous findings^62^ have shown that in the size of the inoculum caused *Lactobacillus casei* to produce more LA and use more lactose.

#### Effect of neutralizing agent

**Fig. 3:**
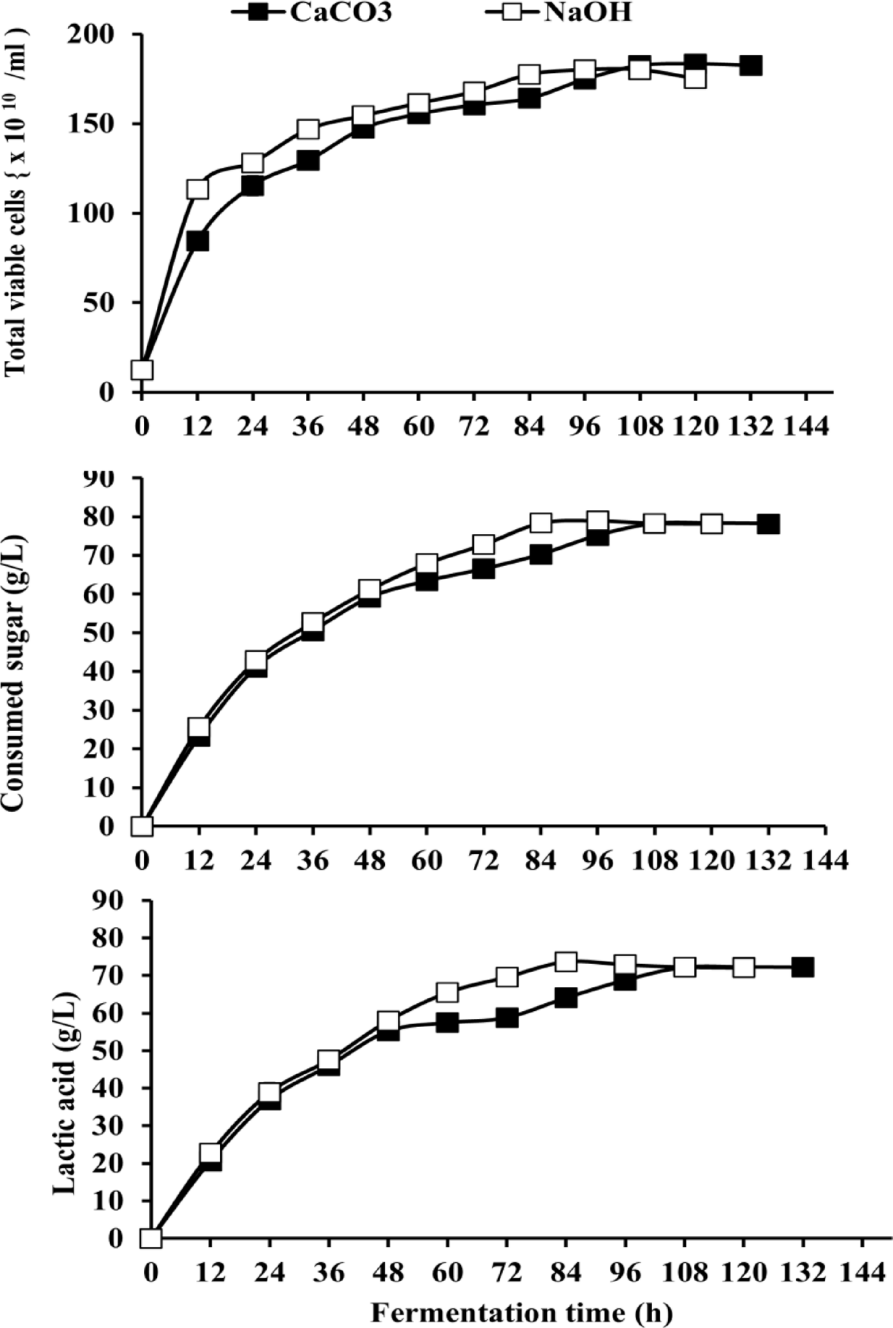
Effect of different neutralizing agents on LA production by *Bacillus licheniformis* OP16-2. The standard deviation is less than the size of symbols if no error bars are seen.

CaCO_3_ and NaOH were utilized as neutralizing agents to evaluate the effect of pH on LA parameters by *B. licheniformis* OP16-2 **(**Fig. 3**).** The findings indicated that using NaOH to neutralize the fermentation media resulted in comparable cell growth and sugar consumption (177.33 ± 2.08 × 10^10^ CFU/mL and 78.3 ± 1.45 g/L, respectively) compared to CaCO_3_ (183.3 ± 2.08 × 10^10^ CFU/mL and 78.3 ± 2.94 g/L, respectively). Additionally, the final concentration of LA was comparable when using CaCO_3_ and NaOH as neutralizing agents (72.2 ± 2.3 g/L and 73.7 ± 1.70 g/L, respectively), which was significantly higher than the fermentation without neutralizing agents (57.4 ± 1.3 g/L). A high LA yield of 0.94 g/g of sugar consumed was achieved with NaOH. Furthermore, LA productivity was increased when NaOH was used for neutralizing the media, reaching 0.87 ± 0.02 g/L/h, compared to 0.60 ± 0.02 g/L/h with CaCO_3_. The NaOH solution had the highest maximal LA productivity (1.88 ± 0.05 g/L/h).

Giraud et al.^63^ found that the growth of LA bacteria is typically hindered by lactate accumulation, which can cause cell membrane collapse, cytosol acidification, or anion buildup within the cell^64,65^. Senthuran et al.^66^ and Timbuntam et al.^67^ reported that high concentrations of soluble substances can have various effects on LA fermentation. Buvukkileci^68^ studied the neutralizing effects of NaOH and CaCO_3_ in LA-production by *Lactobacillus casei* NRRL B-441 and found NaOH to be more effective than CaCO_3_. Several studies^56,69,70^ have reported the use of NaOH to control the pH in LA fermentation processes. NaOH proved superior to CaCO_3_ not only due to better fermentation parameters but also because it avoids the formation of gypsum waste from CaCO_3_ supplementation^18^. Conversely, Oliveira et al.^71^ found that the pH value was decreased to 5.0 within 12 h., of using CaCO_3_ and remained there, resulting in ineffective LA production. In comparison to other neutralizers, Coelho et al.^72^ found that Ca(OH)_2_ increased LA production by 10–15%.

#### Effect of different concentrations of YE

For LA fermentation to be cost effective, nitrogen additives are crucial^73^. Table 7 shows that the highest cell growth in term of total viable cells increased from 177.6 ± 1.5 × 10^10^ CFU/mL at 5 g/L YE to 171.7 ± 2.0 × 10^10^ CFU/mL when YE was excluded entirely. Sugar consumption is slightly decreased from 78.2 ± 1.6 g/L at 5 g/L to 76.8 ± 0.7 g/L without YE. However, the final LA-concentration was increased from 71.0 ± 1.1 g/L to 73.6 ± 1.7 g/L with 5 g/L YE and slightly decreased to 71.0 ± 1.1 g/L when YE was removed, indicating that the LA-production is not significantly affected by YE supplementation. The highest LA yield and productivity were 0.94 g/g and 0.88 ± 0.02 g/L/h, respectively.

**Table 7:** Effect of various concentrations of yeast extract on LA fermentation parameters by *B. licheniformis* OP16-2.

Yeast extract (g/L)	Total Viable Count (×10^10^) CFU/mL	Consumed sugar (g/L)	LA conc. (g/L)^a^	Y_LA_ (g/g)^b^	*P*_LA_ (g/L/h)^c^	Max *P*_LA_ (g/L/h)^d^ at the indicated time
5	177.6 ± 1.5 a	78.2 ± 1.6 a	73.6 ± 1.7 a	0.94 ± 0.00 a	0.88 ± 0.02 a	1.9 ± 0.06(12)
3	175 ± 4.5 a	76.3 ± 1.5 a	71.3 ± 1.34 a	0.93 ± 0.01 a	0.85 ± 0.01 a	1.8 ± 0.11(12)
2	171.7 ± 2.1 a	76.7 ± 0.6 a	70.9 ± 1.1 a	0.92 ± 0.00 a	0.84 ± 0.01 a	1.8 ± 0.08(12)
0	171.7 ± 2.0 a	76.8 ± 0.7 a	71.0 ± 1.1 a	0.92 ± 0.00 a	0.85 ± 0.01 a	1.8 ± 0.08(12)

^a^Maximum lactic acid concentration at 84 h, ^b^Lactic acid yield, ^c^Lactic acid productivity at the end of fermentation time, ^d^Maximum lactic acid productivity at the indicated time. Different lower-case letters in the same column are significantly different by post hoc Tukey’s test at *p* < 0.05; values in the same column with the same letter are not significantly different.

Previous research highlighted the effectiveness of CSW as a fermentation substrate by detailing its acidity and the concentrations of sugars, sulfites, free amino acids, reducing sugars, and nitrogen^74^. CSW has been utilized to provide additional nutrients in growth media and fermentation processes^75^. CSW was selected as a YE substitute for the fermentation of syngas due to its cost-effectiveness and higher nutrient content^76,77^.

#### Effect of pH values

The accumulation of LA during the fermentation process leads to a decrease in pH^78^. To investigate the effect of initial pH values on LA production by *B. licheniformis* OP16-2, fermentation processes were conducted at pH values of 8.0, 8.5, 9.0, and 9.5, while maintaining all previously optimized parameters. Table 8 presents the profiles and LA fermentation parameters for LA production at these pH values. Cell-growth increased from 171.6 ± 2.08 × 10¹⁰ CFU/mL at pH 8.0 to a peak of 177.6 ± 1.52 × 10¹⁰ CFU/mL at pH 8.5. However, the total viable count was decreased from 172 ± 1.0 × 10¹⁰ CFU/mL at pH 9.0 to the lowest value of 170 ± 4.5 × 10¹⁰ CFU/mL at pH 9.5. Conversely, sugar consumption by the OP16-2 strain was increased from its lowest value of 76.73 ± 0.64 g/L at pH 8.0 to a highest value of 78.16 ± 1.5 g/L at pH 8.5, then decreased to 74.9 ± 2.06 g/L at pH 9.5. LA production followed a similar pattern, with the final LA concentration rising from 70.8 ± 1.05 g/L at pH 8.0 to a maximum of 73.6 ± 1.73 g/L at pH 8.5, while it decreased to a minimum of 67.2 ± 4.5 g/L at pH 9.5. Meanwhile, the LA yield ranged from 0.89 to 0.94 g/g of sugars consumed at pH values of 8.0 to 9.5, with the highest yield of 0.94 g/g at pH 8.5.

**Table 8:** Effect of various pH values on LA fermentation parameters by *B. licheniformis* OP16-2.

pH values	Total Viable Count (×10^10^) CFU/mL	Consumed sugar (g/L)	LA conc. (g/L)^a^	Y_LA_ (g/g)^b^	*P*_LA_ (g/L/h)^c^	Max *P*_LA_ (g/L/h)^d^ at the indicated time
8.0	171.6 ± 2.08 ab	76.73 ± 0.64 a	70.86 ± 1.05 a	0.92 ± 0.00 a	0.84 ± 0.01 a	1.83 ± 0.08(12)
8.5	177.6 ± 1.52 a	78.16 ± 1.5 a	73.63 ± 1.73 a	0.94 ± 0.00 a	0.87 ± 0.02 a	1.88 ± 0.05(12)
9.0	172 ± 1.0 ab	76.8 ± 0.66 a	71.33 ± 1.66 a	0.92 ± 0.01 a	0.84 ± 0.02 a	1.83 ± 0.08(12)
9.5	170 ± 4.5 b	74.9 ± 2.06 a	67.23 ± 4.5 a	0.89 ± 0.03 a	0.80 ± 0.05 a	1.73 ± 0.02(12)

^a^Maximum lactic acid concentration at 84 h, ^s^Lactic acid yield, ^c^Lactic acid productivity at the end of fermentation time, ^d^Maximum lactic acid productivity at the indicated time. Different lower-case letters in the same column are significantly different by post hoc Tukey’s test at *p* < 0.05; values in the same column with the same letter are not significantly different.

At pH 8.5, LA productivity was increased to 0.87 ± 0.02 g/L/h, but at pH 9.5, it dropped to at 0.80 ± 0.05 g/L/h. The maximum LA productivity was ranged 1.73 ± 0.02 to 1.88 ± 0.05 g/L/h, with pH 8.5 exhibiting the highest value of 1.88 ± 0.05 g/L/h.

### Optimization by RSM in batch fermentation

To determine the optimum variables for achieving the maximum LA productivity, LA concentration, and LA yield, RSM was performed.

#### RSM for LA concentrations

##### ANOVA for main and interaction effects

A factorial analysis of variance (ANOVA) was employed to ascertain how the concentration of LA changed in response to different situations. The *p*-values and R-squared (r²) values were used to assess the model’s significance (*p* ≤ 0.05) and its fit to the experimental data. The data in Table 9 show the significance (*p*-value), model coefficients, and main and two-way interaction effects for the entire 25 full factorial CCD for LA production (g/L). The ANOVA model also investigated the interaction effects. When a factor’s reaction changes from low to high levels in response to the level of another factor, this is known as a factor interaction. Our findings demonstrate that the effects of optimizing five distinct variables result in favorable agreement with the experimental findings (OFAT).

**Table 9:** ANOVA model results to examine variations in *B. licheniformis* OP16-2 lactic acid production (g/L) from CSW in response to various stimuli. Significant variations are indicated in bold. (r^2^ = 98.1%).

Source	df	Adj SS	Adj MS	F-Value	*p*-Value
Model	19	24703.4	1300.2	115.4	0.000
Linear (Main Effect)	5	1848.4	369.7	32.8	**0.000**
Sugar Conc.	1	79.9	79.9	7.09	**0.011**
Temp.	1	82.2	82.2	7.29	**0.010**
Incula. Size	1	1677.0	1677.0	148.8	**0.000**
pH	1	3.2	3.2	0.28	0.598
YE	1	6.2	6.2	0.55	**0.464**
Square	4	22539.2	5634.8	500.1	**0.000**
Sugar Conc. × Sugar Conc.	1	10348.9	10348.9	918.6	**0.000**
Temp. ×Temp.	1	4567.7	4567.7	405.4	**0.000**
Incula. Size × Incula. Size	1	5387.3	5387.3	478.1	**0.000**
pH × pH	1	9039.1	9039.1	802.3	**0.000**
2-Way Interaction	10	315.8	31.6	2.80	0.009
Sugar Conc. × Temp.	1	22.2	22.2	1.97	0.168
Sugar Conc. × Incula. Size	1	259.9	259.9	23.0	**0.000**
Sugar Conc. × pH	1	0.1	0.1	0.01	0.942
Sugar Conc. ×YE	1	0.0	0.0	0.00	1.000
Temp. × Incula. Size	1	33.6	33.6	2.98	0.091
Temp. × pH	1	0.0	0.0	0.00	0.975
Temp. × YE	1	0.0	0.0	0.00	1.000
Incula. Size ×pH	1	0.0	0.0	0.00	0.975
Inocula. Size ×YE	1	0.0	0.0	0.00	1.000
pH ×YE	1	0.0	0.0	0.00	1.000
Error	42	473.2	11.3		
Lack-of-Fit	30	471.4	15.7	104.6	0.000
Pure Error	12	1.8	0.2		
Total	61	25176.6			

Except for sugar concentrations and inoculum sizes, there was no significant interaction between any of the two-way terms. The relevance of all squared terms indicates a nonlinear link between the variables and LA concentration. The produced LA may be represented by the following equation, as at the 95% confidence level, every other impact was significant. Regression in uncoded units using Eq. (1):

$$\begin{array}{*{20}{l}}{With\;yeast\;extract\left(LA \; {g/L} \right) = - 4645 + \;1.685\;Sug.conc + \;32.95\;Temp. + \;41.96\;Inoculum\;size + \;854.0\;pH}\\{ - \;0.014946\;Sug.Conc \times Sug.Conc - \;0.3575\;Temp. \times Temp.}\\{ - \;1.5529\;Inoculum\;size \times Inoculum\;size - \;50.29\;pH \times pH}\\{ + \;0.00555\;Sug.conc \times Temp. + \;0.03800\;Sug.concxInoculum\;size}\\{ + \;0.0029\;Sug.conc \times pH - \;0.0820\;Temp. \times Inoculum\;size + \;0.007\;Temp.xpH}\\{ - \;0.015\;Inoculum\;sizexpH}\end{array}$$

$$\begin{array}{*{20}{l}}{{Without}\;{yeast\;extract}\;LA\left( {g/L} \right) = - 4646 + \;1.685\;Sug.conc + \;32.95\;Temp. + \;41.96\;Inoculum\;size + \;854.0\;pH}\\{ - \;0.014946\;Sug.Conc \times Sug.Conc - \;0.3575\;Temp. \times Temp.}\\{ - \;1.5529\;Inoculum\;size \times Inoculum\;size - \;50.29\;pH \times pH}\\{ + \;0.00555\;Sug.conc \times Temp. + \;0.03800\;Sug.conc \times Inoculum\;size}\\{ + \;0.0029\;Sug.conc \times pH - \;0.0820\;Temp. \times Inoculum\;size + \;0.007\;Temp. \times pH}\\{ - \;0.015\;Inoculum\;size \times pH}\end{array}$$

Using **Eq. (1)**, it is possible to predict LA production based on initial pH, temperature, inoculum size, initial CSW-concentrations, and their interactions, both with and without YE.

**Fig. 4:**
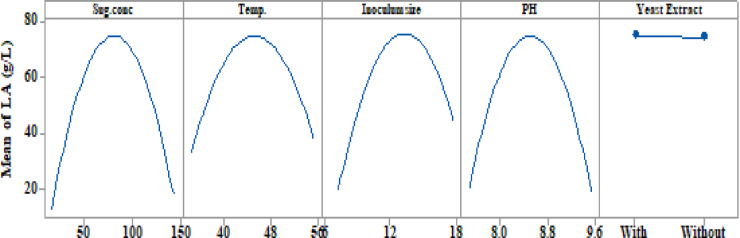
Main effects plots explain the changes in LA production by *B. licheniformis* OP16-2 between low and high levels of each factor.

The findings of the prediction analysis are shown in the major effect plots **(**Fig. 4**)**, which show the average variances between high and low levels for each factor. The data showed that whereas temperature, inoculum sizes, pH values, and sugar concentration all significantly varied between low- and high- concentrations of LA, YE had no discernible effect on LA concentrations.

##### Normal probability plots and Pareto charts

**Fig. 5:**
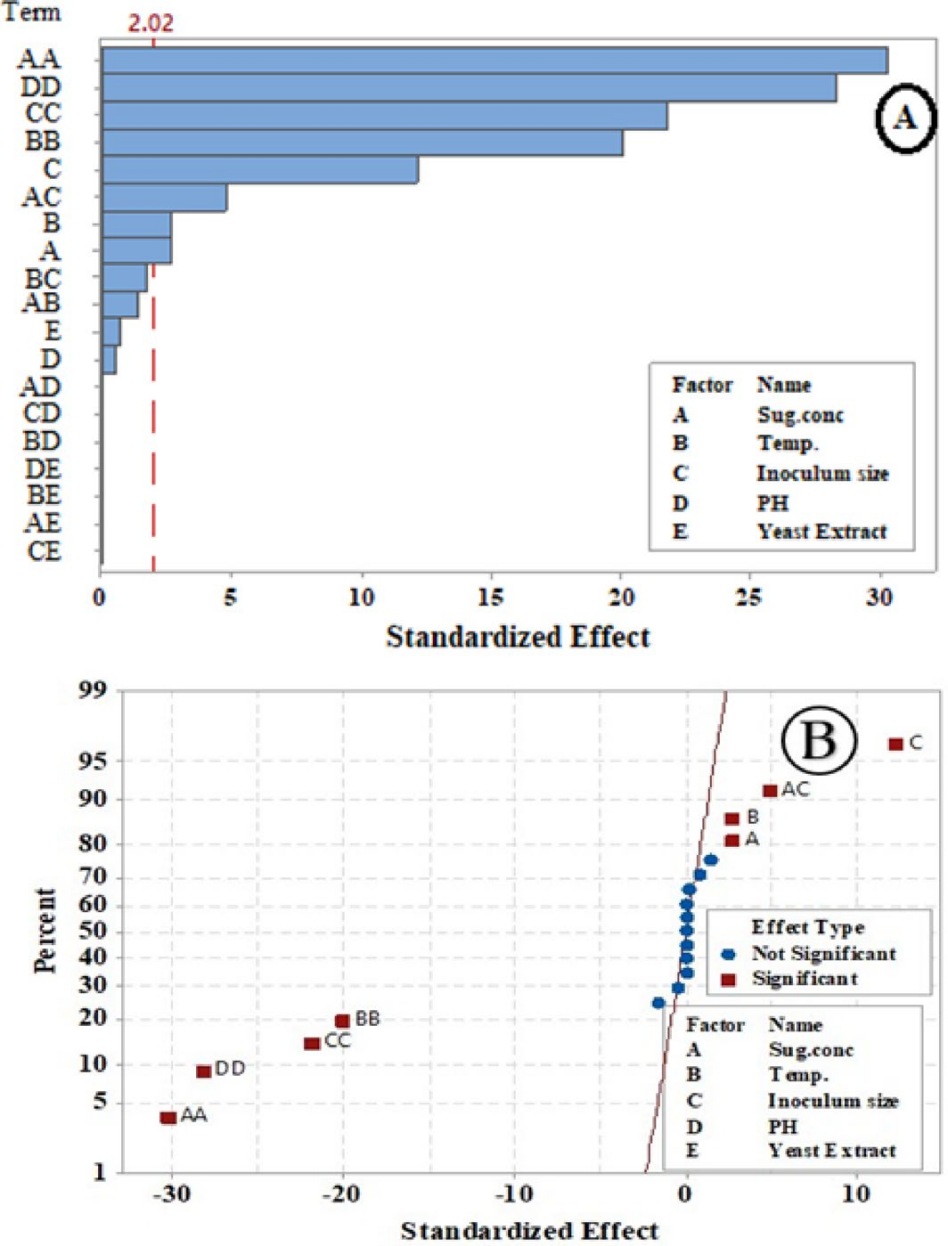
Pareto chart (**A**) and normal probability plot (**B**) of LA concentration (g/L) by *B. licheniformis* OP16-2.

The relative relevance and significance of the main and interaction effects on LA fermentation after 84 h. were assessed using the Pareto chart (Fig. 5A). With a *p*-value of less than 0.05, the graphic indicated that the primary impacts of temperature (B), inoculum size (C), sugar concentration (A), and their interactions (AA, BB, CC, DD, and AC) exceeded the reference line. The lengths of the columns show the proportional importance of each phrase.

The main factors of inoculum size (C), sugar concentration (A), and temperature (B), along with the interaction between sugar concentration and inoculum size (AC), are significantly distant from the fitted line, indicating a substantial impact on LA concentration (g/L). Their positive effect is shown by their position on the right side of the graph (Fig. 5B). Sugar concentration notably influenced lactic acid concentration (g/L), and several scientists have observed that initial sugar concentrations increase LA concentration up to a certain point^56,57,79^. Sugar plays a crucial role in the cost-effective production of lactic acid^80^. Conversely, the interactions between all terms (AA, BB, CC, and DD) are positioned to the left of the fitted line, indicating a significant negative impact on LA concentration (Fig. 5B).

##### Contour and surface plots of LA production (g/L)

**Fig. 6:**
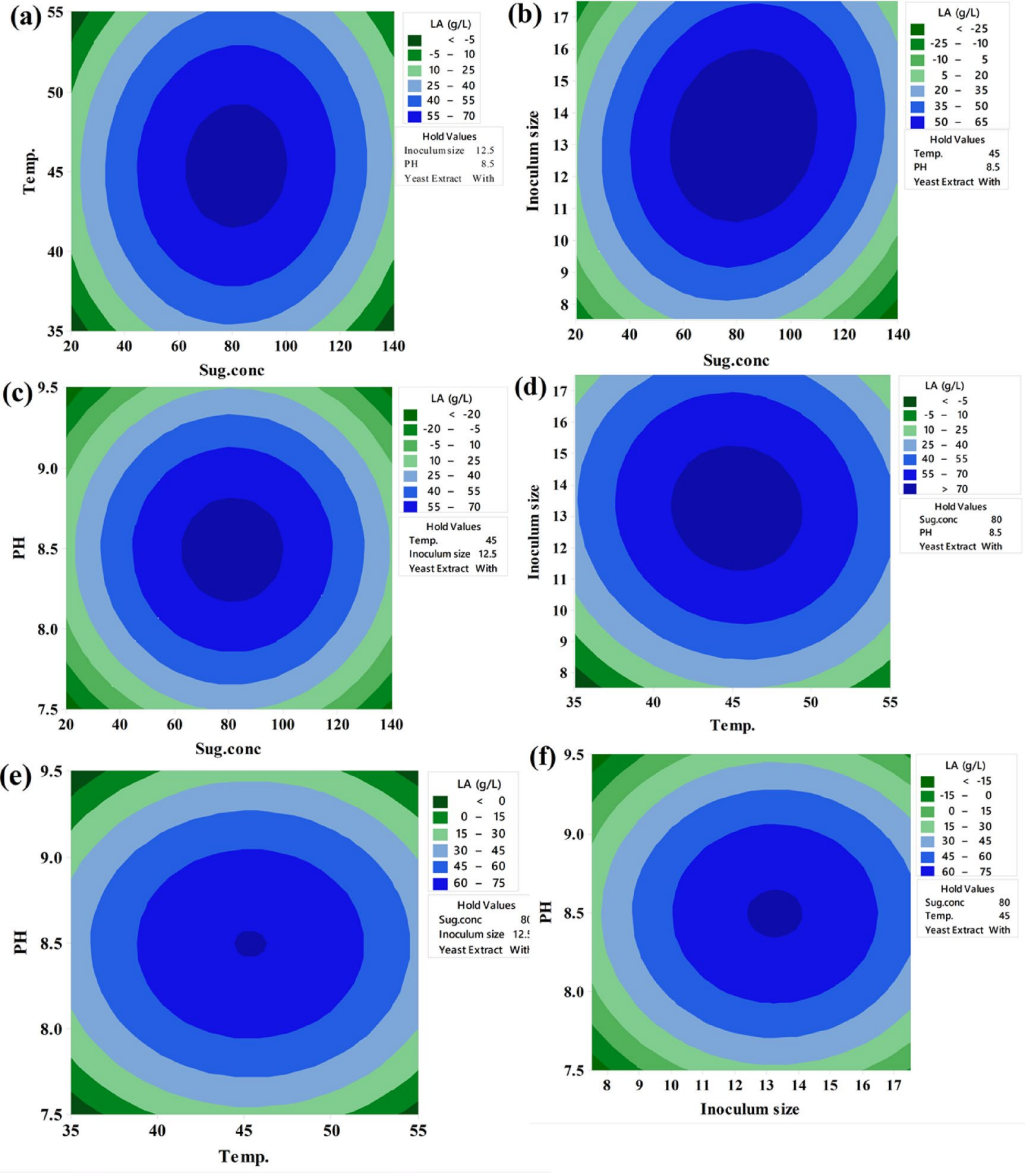
The predicted reaction surface’s contours for LA concentration (g/L) by *B. licheniformis* OP16-2.

**Fig. 7:**
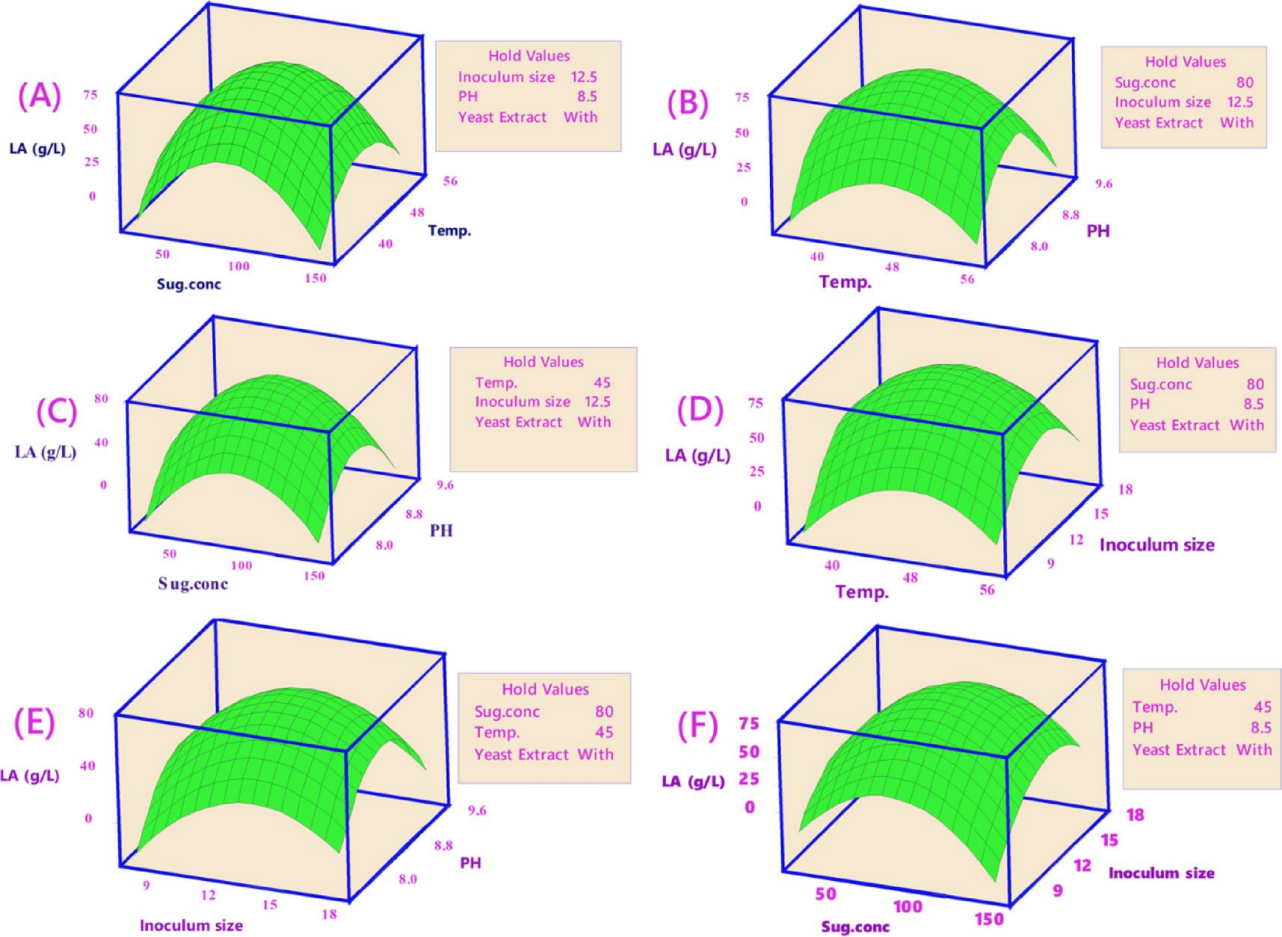
Surface plots for LA concentration (g/L) from CSW by *B. licheniformis* OP16-2.

Keeping the third component constant, contour plots were created to visually represent the increasing LA concentration (g/L) based on the significance of the main and two-way interaction effects. These two-dimensional graphs connect points with the same response value to form contour lines, ranging from minimum to maximum response values (Fig. 6). Surface-wireframe plots, which are three-dimensional graphs, illustrate the relationship between the response and each pair of interdependent process factors. These 3D grid plots display the optimal peaks, indicating the highest response values. Surface plots of the response functions facilitate a better understanding of the main, square, and interaction effects (Fig. 7).

A typical simple maximum pattern was displayed by the contour and surface plots of LA production between the same interactions (Figs. 6 and 7A), where the LA was increased to more than 70 g/L at the interaction between levels of sugar ranging from 70 to 90 g/L and temperature ranging from 40 to 50 °C., while maintaining constants for pH, inocula size, and YE supplementation.

A typical highest pattern was observed in the contour and surface plots of LA production for the same interactions (Figs. 6 and 7). LA concentration was increased to over 70 g/L when levels of sugar ranged from 70 to 90 g/L and temperature ranged from 40 to 50 °C, while keeping other factors constant (pH, inoculum size, and supplementation of YE) (Figs. 6 and 7A). Similarly, LA concentration was increased with the interaction between inoculum size (8–13%) and sugar concentrations (70–90 g/L), while other factors remained constant (Figs. 6 and 7F). LA concentration also exceeded 60 g/L at the interaction between pH and sugar concentrations, with different factors constant (Figs. 6 and 7C). When the temperature was around 45 °C, and the inoculum size ranged from 13 to 15%, LA concentration peaked at 72 g/L (Figs. 6 and 7D). Additionally, LA concentration reached 73 g/L at the interaction between pH values (7.0–8.0) and inoculum size (13–15%), while keeping other factors constant (Figs. 6 and 7E). Our findings showed that key factors, particularly sugar concentration, had a significant influence on lactic acid production. Similarly, several researchers observed that initial sugar concentrations increased LA concentration up to a certain point^56,57,79^. Sugar is a key factor in the cost-effective production of LA^80^.

#### RSM for enhanced LA yield (g/g)

##### ANOVA for main and interaction effects

**Fig. 8:**
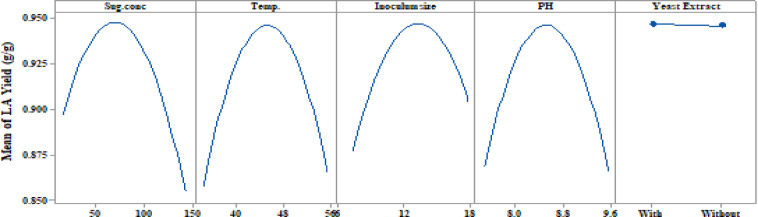
Plots of main effects describe how the LA yield (g/g) of CSW by *B. licheniformis* OP16-2 varies between low and high concentrations of each factor.

To identify variations in LA yield (g/g) under different conditions, an ANOVA was employed for the full 2^5^-factorial CCD. Table 10 lists the main effects, square effects, and two-way interaction effects, along with their corresponding significance levels (*p*-values). Except for YE, the data in Table 10, which are graphically depicted in Fig. 8, reveal a nonlinear connection between the parameters and LA yield (g/g). All bold terms are significant at a 95% confidence level.

**Table 10:** ANOVA-model results to check for variations in LA yield (g/g). (r^2^ = 92.5%).

Source	df	Adj SS	Adj MS	F-Value	*p*-Value
Model	19	24703.4	1300.2	115.41	0.000
Linear (Main Effect)	19	0.060026	0.003159	27.34	**0.000**
Sugar Conc.	5	0.007041	0.001408	12.19	**0.000**
Temp.	1	0.004901	0.004901	42.41	**0.000**
Inoculum Size	1	0.000145	0.000145	1.25	0.269
pH	1	0.001970	0.001970	17.05	**0.000**
YE	1	0.000013	0.000013	0.11	0.737
Square	1	0.000012	0.000012	0.10	0.750
Sugar Conc. × Sugar Conc.	4	0.048376	0.012094	104.6	**0.000**
Temp. × Temp.	1	0.014447	0.014447	125.0	**0.000**
Incula. Size × Incula. Size	1	0.021126	0.021126	182.8	**0.000**
pH × pH	1	0.009208	0.009208	79.6	**0.000**
2-Way Interaction	1	0.018373	0.018373	159.0	0.000
Sugar Conc. × Temp.	10	0.004609	0.000461	3.99	**0.001**
Sugar Conc. × Incula. Size	1	0.000229	0.000229	1.98	0.166
Sugar Conc. × pH	1	0.003797	0.003797	32.8	**0.000**
Sugar Conc. × YE	1	0.000059	0.000059	0.51	0.477
Temp. × Incula. Size	1	0.000000	0.000000	0.00	0.972
Temp. × pH	1	0.000322	0.000322	2.79	0.102
Temp. × YE	1	0.000001	0.000001	0.01	0.931
Incula. Size × pH	1	0.000000	0.000000	0.00	0.973
Inocula. Size × YE	1	0.000196	0.000196	1.70	0.200
pH × YE	1	0.000003	0.000003	0.03	0.866
Error	1	0.000000	0.000000	0.00	0.997
Lack-of-Fit	42	0.004853	0.000116		
Pure Error	30	0.004648	0.000155	9.04	0.000
Total	61	0.064879			

Except for the relationships between sugar content and temperature and pH, none of the two-way terms interacted significantly. All squared terms, however, were significant, suggesting that the variables and LA yield had a nonlinear relationship. At a 95% confidence level, all additional bold effects were significant.

The average deviations between high and low levels for each factor were displayed in the major effect plots **(**Fig. 8**)** to show the results of the prediction analysis. The findings demonstrated that while YE had no discernible effect on LA yield, temperature, sugar content, inoculum sizes, and pH values all had notable variations between their high and low levels.

##### The Pareto charts and normal probability plots

**Fig. 9:**
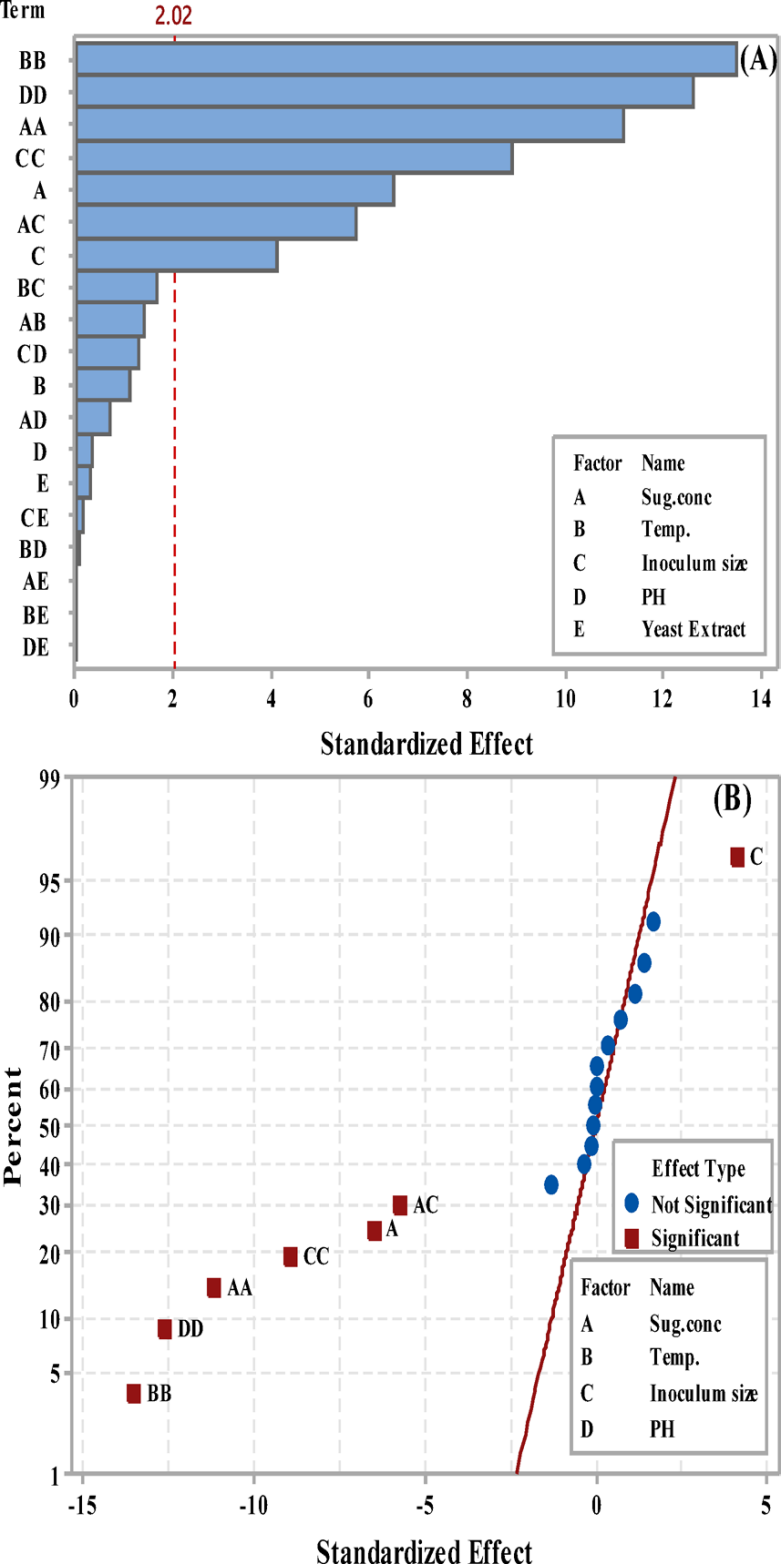
Pareto-chart (**A**) and normal-probability plot (**B**) of standardized effects in LA yield (g/g).

The relative impact and significance of the main interaction effects were assessed using the Pareto chart **(**Fig. 9A**)**, which focused on the factors influencing LA yield after 84 h.

The main effects of sugar concentrations (A), inoculum sizes (C), and their interactions (AA, DD, CC, BB, AC) extend beyond the reference line in the Pareto-chart of standardized effects for LA yield **(**Fig. 9A**)**, indicating a significant impact at a *p*-value of ≤ 0.05. The column length represents the relative importance of each term. Normal probability plots show whether an effect is positive or negative on the response. The reaction increases when the factor increases, which is known as a positive effect. The opposite is true for a negative effect. Each effect has its own point on the plot; variables with no discernible influence are shown by points near the fitted line (where effects are zero), while the actual term effect is represented by points far from the line. The graph’s right side displays a positive effect. In contrast, the main effects, such as inoculum size (C), are distant from the fitted line, indicating a substantial and significant impact on LA yield (Fig. 9B). Conversely, all terms and interactions to the left of the fitted line indicate a significant negative effect on LA yield (Fig. 9B**).**

##### Contour plots of LA yield (g/g)

**Fig. 10:**
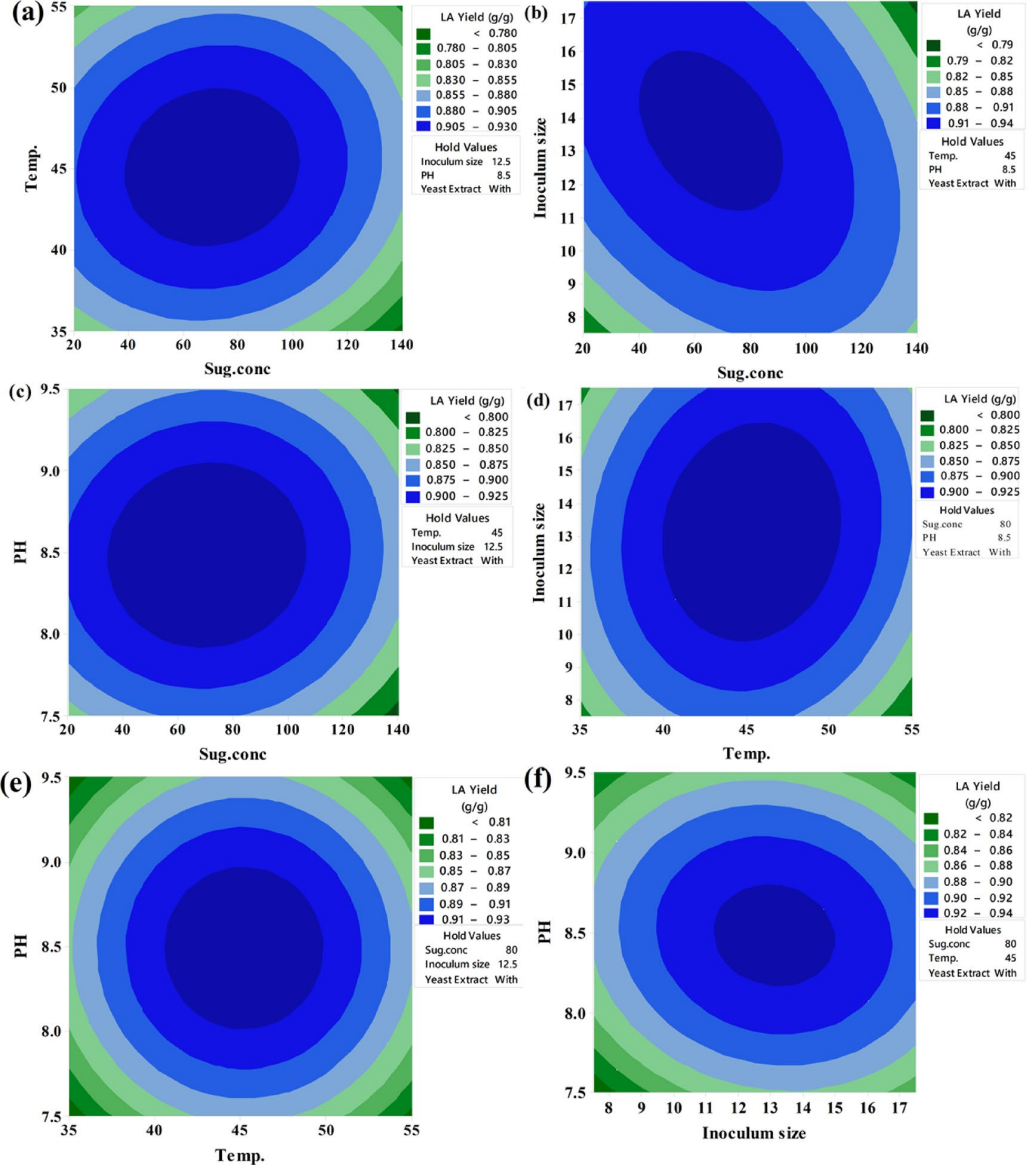
The predicted reaction surface’s contours for LA yield (g/g) from CSW by *B. licheniformis* OP16-2.

Contour plots were developed to visually suit the increasing LA yield of each two-factor while maintaining the third factor, which is not displayed in the plot, based on the significance of the main and two-way interaction effects. The contour plot is a two-dimensional graph where all points from each element with the same response value are joined to form contour lines that go from the lowest to the highest response values (Fig. 10).

##### Surface plots for LA yield (g/g)

**Fig. 11:**
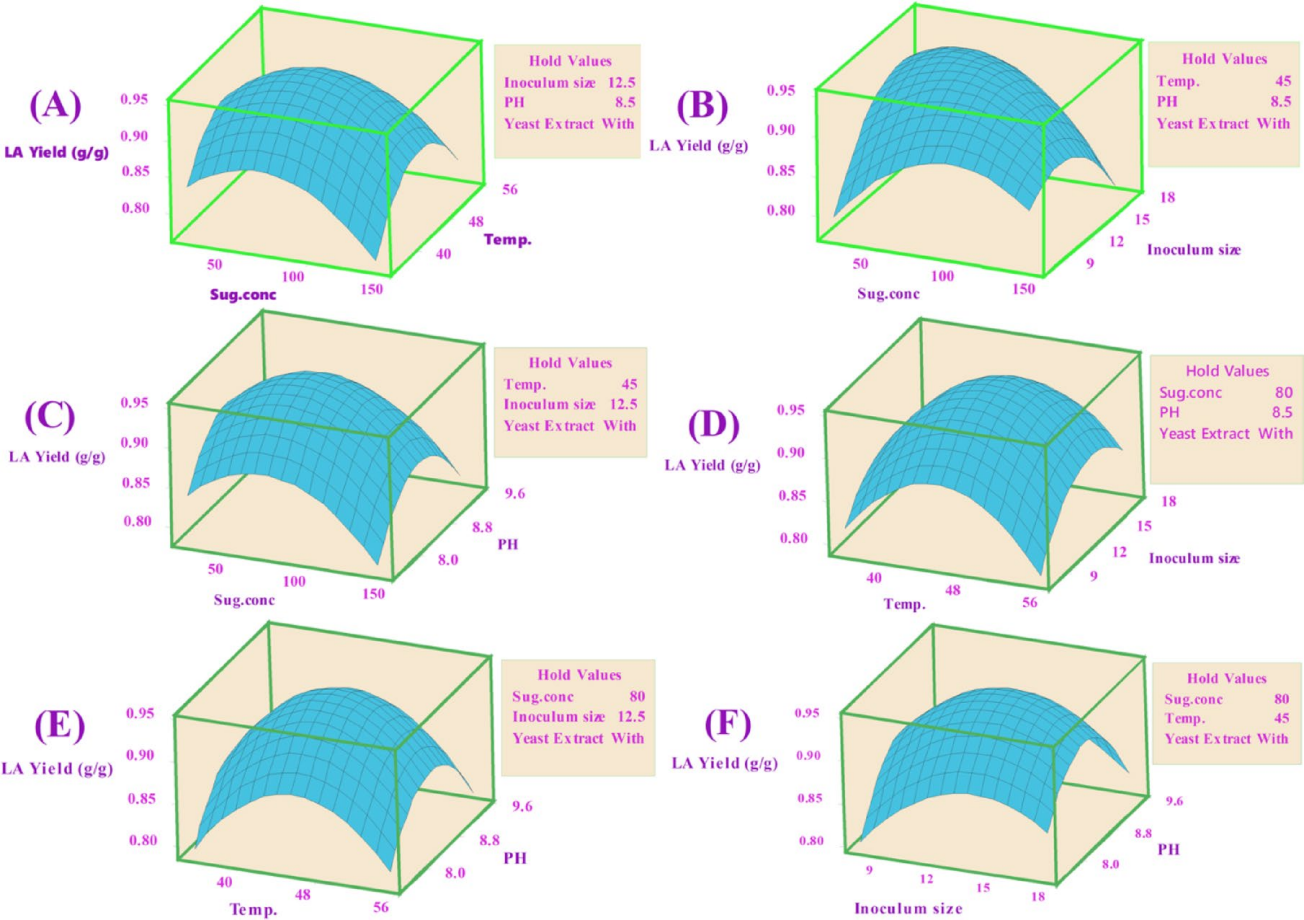
Surface plots for LA yield (g/g) from CSW by *B. licheniformis* OP16-2.

Figure 11 shows the surface plots of LA yield between the same interactions, where, at sugar levels ranging from 40 to 70 g/L and temperatures varying from 40 to 55 °C, the LA yield increased to 0.92 g/g (Fig. 11A). The LA yield also increased to 0.92 for inoculum sizes between (7.5–13%) and sugar concentrations between (40–70 g/L) (Fig. 11B). Additionally, for pH values between 8.0 and 9.0, with sugar concentrations between 40 and 70 g/L, the LA yield increased to 0.92 g/g (Fig. 11C). The LA production increased to 0.92 g/g when the temperature was about 45 °C and the inoculum sizes were about (12.5%) (Fig. 11D). The LA yield also reached 0.92 g/g at a pH value ranging (from 8.0 to 9.0) with a temperature of about 45 °C and inoculum sizes of around (12.5%) (Fig. 11E and F).

#### RSM for LA productivity (g/L/h)

##### Main and interaction effects (ANOVA)

For all 2^5^ factorial designs, the data displayed in Table 11 included the main, interaction effect, model coefficients, standard deviation of each coefficient, and probability. The significance of the regression coefficients was verified using a student’s t-test. A 95% confidence level indicated that all main and square effects were very significant. To match the statistical model, however, the model showed an adjusted R-squared correlation coefficient of 98.1%.

**Table 11:** ANOVA model results to check for variations in LA productivity. (R^2^ = 98.1%).

Source	Df	Adj SS	Adj MS	F-Value	*p*-Value
Model	19	2.68049	0.14108	115.41	0.000
Linear (Main Effect)	5	0.20056	0.04011	32.81	**0.000**
Sugar Conc.	1	0.00867	0.00867	7.09	**0.011**
Temp.	1	0.00892	0.00892	7.29	**0.010**
Incula. Size	1	0.18197	0.18197	148.86	**0.000**
pH	1	0.00035	0.00035	0.28	0.598
YE	1	0.00067	0.00067	0.55	0.464
Square	4	2.44566	0.61142	500.16	0.000
Sugar Conc. × Sugar Conc.	1	1.12292	1.12292	918.6	**0.000**
Temp. × Temp.	1	0.49563	0.49563	405.4	**0.000**
Incula. Size × Incula. Size	1	0.58456	0.58456	478.1	**0.000**
pH × pH	1	0.98081	0.98081	802.3	**0.000**
2-Way Interaction	10	0.03427	0.00343	2.80	0.009
Sugar Conc. × Temp.	1	0.00241	0.00241	1.97	0.168
Sugar Conc. × Incula. Size	1	0.02820	0.02820	23.07	**0.000**
Sugar Conc. × pH	1	0.00001	0.00001	0.01	0.942
Sugar Conc. × YE	1	0.00000	0.00000	0.00	1.000
Temp. × Incula. Size	1	0.00365	0.00365	2.98	0.091
Temp. × pH	1	0.00000	0.00000	0.00	0.975
Temp. × YE	1	0.00000	0.00000	0.00	1.000
Incula. Size × pH	1	0.00000	0.00000	0.00	0.975
Inocula. Size × YE	1	0.00000	0.00000	0.00	1.000
pH × YE	1	0.00000	0.00000	0.00	1.000
Error	42	0.05134	0.00122		
Lack-of-Fit	30	0.05115	0.00170	104.6	0.000
Pure Error	12	0.00020	0.00002		
Total	61	2.73183			

**Fig. 12:**
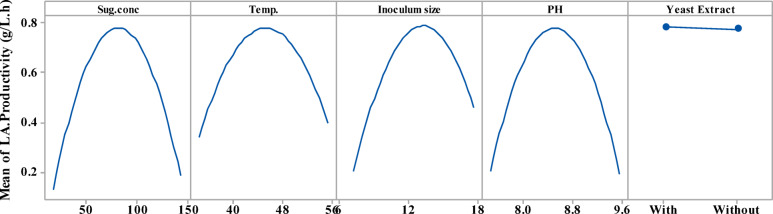
Main effects plots explain the changes in LA productivity (g/L/h) from CSW by *B. licheniformis* OP16-2.

The main effect plots **(**Fig. 12**)** display the average variances between high and low levels for each factor, representing the results of the prediction analysis. YE did not impact LA productivity, whereas temperature, pH values, sugar concentration, and inoculum sizes showed significant differences between their low and high levels.

##### The Pareto-charts and normal -probability plots

**Fig. 13:**
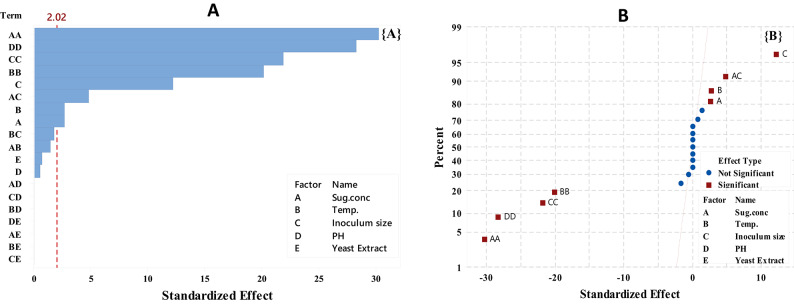
Pareto-Chart (**A**) and normal-probability plot (**B**) of standardized effects in LA productivity (g/L/h) from CSW by *B. licheniformis* OP16-2.

The main effects of sugar-levels (**A**), Temp. (**B**), inoculum sizes (C), and their interactions (AA, BB, CC, DD, AC) extend beyond the reference line, indicating a significant impact at a *p*-value of ≤ 0.05. The column length represents the relative importance of each term **(**Fig. 13A**).**

.

LA- productivity (g/L/h) is strongly impacted by the primary parameters of inoculum size (C), sugar content (A), Temp (B), and their interaction (AC), all of which are somewhat far from the fitted line. The graph’s right side displays their beneficial contribution (Fig. 13B). On the other hand, interactions between pH (DD), inoculum size (CC), temperature (BB), and sugar concentration (AA) are located to the left of the fitted line, suggesting a substantial adverse impact on LA productivity (Fig. 13B). Previous observations have shown that inoculum size significantly increases LA productivity. The optimal inoculum size for *E. faecalis* KY-072975’s LA-fermentation was 5% (*v/v*)^81^, resulting in a progressive increase in LA productivity. For *Lactobacillus amylophilus* GV6, the best inoculum concentration for LA productivity was 10% (*v/v*)^82^. *Lacticaseibacillus* casei NBIMCC 1013 achieved the highest LA production rate with an inoculum size of 2 to 4% (*v/v*)^83^.

##### Contour and surface plots of LA productivity

**Fig. 14:**
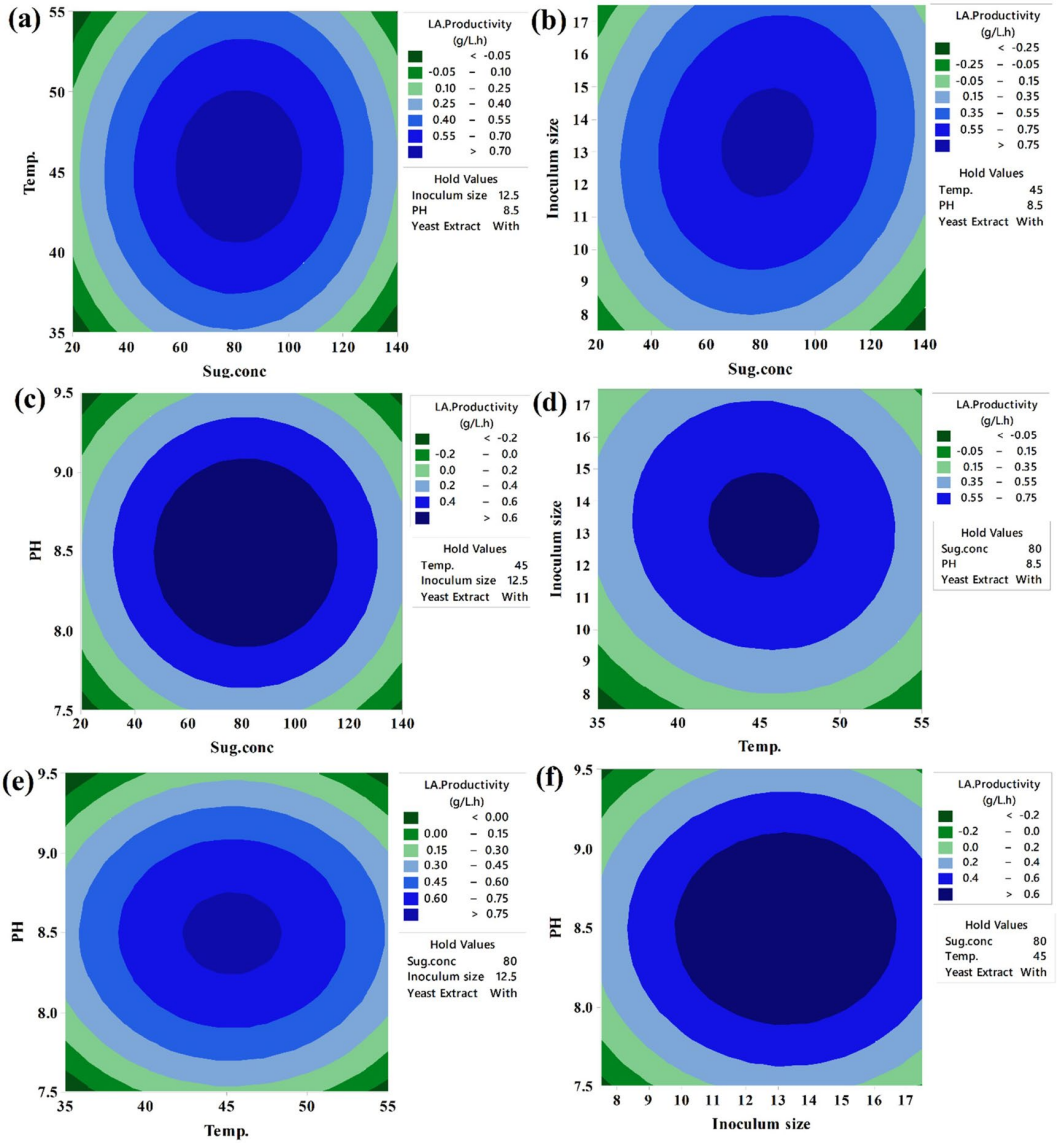
The predicted reaction surface’s contours for LA productivity (g/L/h) from CSW by *B. licheniformis* OP16-2.

**Fig. 15:**
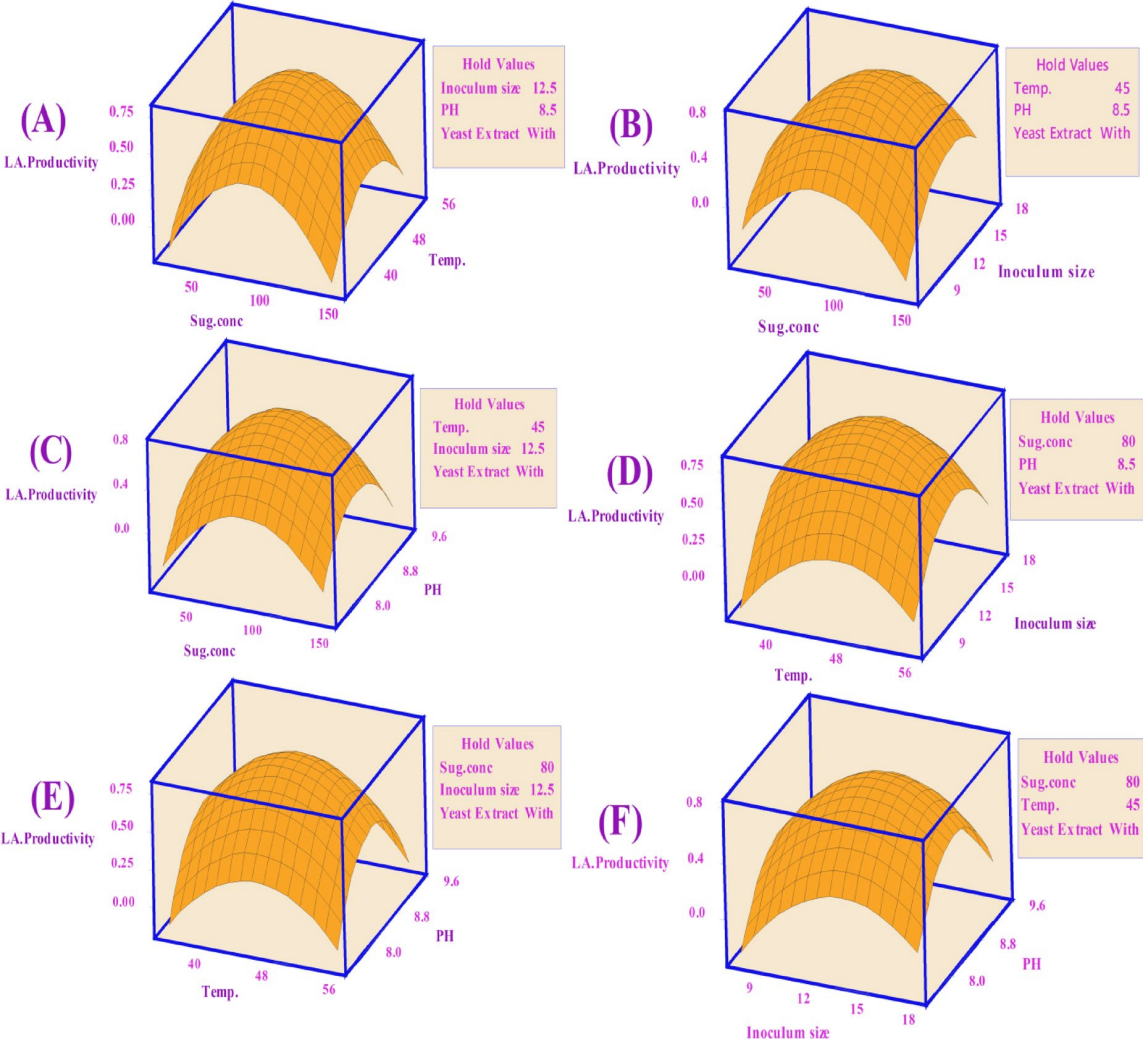
Surface plots for LA productivity (g/L/h) from CSW by *B. licheniformis* OP16-2.

**Fig. 16:**
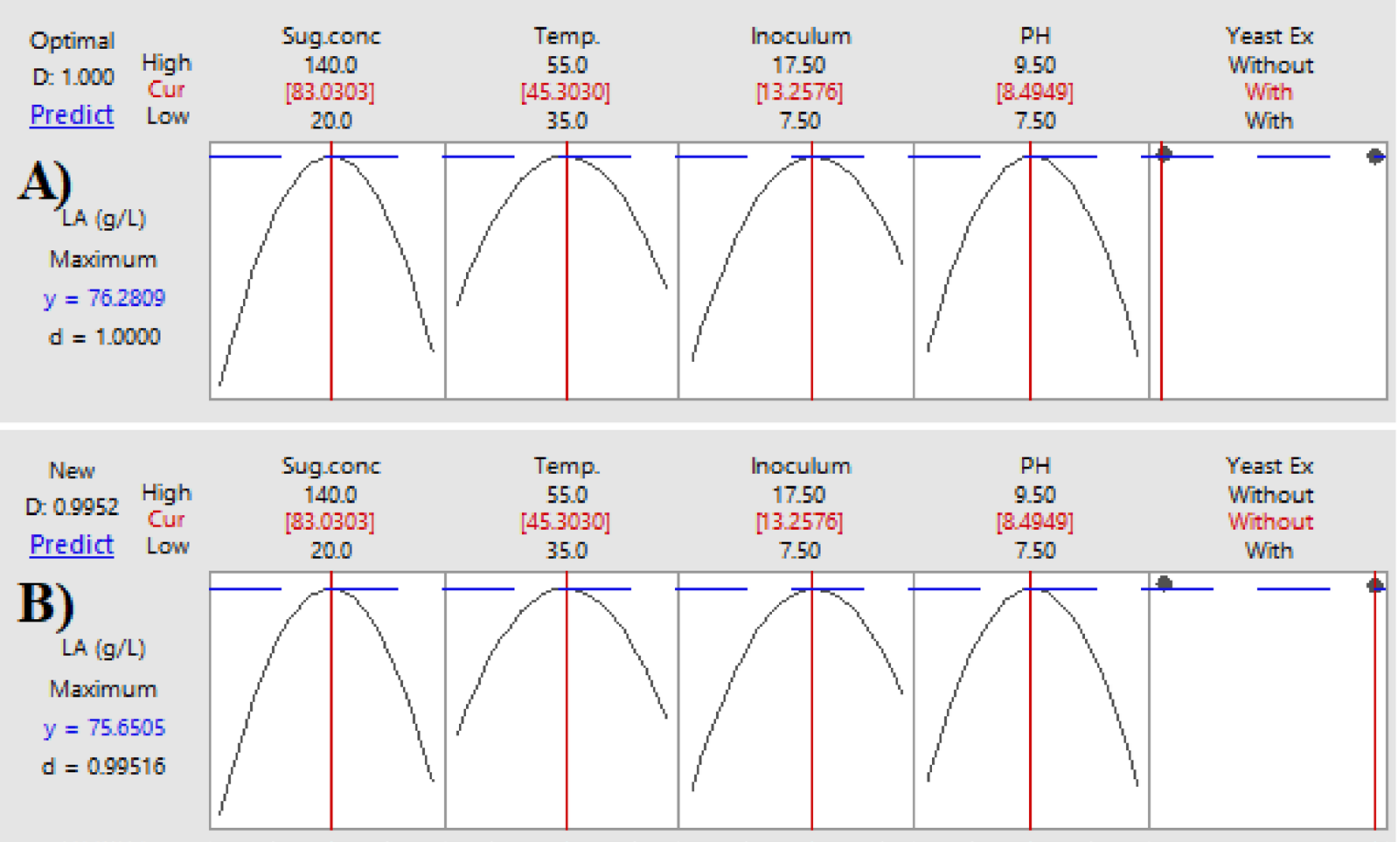
The factors’ effects on the anticipated responses (y) are displayed by the optimization curves, including maximum LA concentration (g/L) with YE (**A**) and LA production without YE (**B**) at low and high levels. The optimum factor settings (Cur) were predicted to have a composite desirability (d) of 1.000 (100%) with YE and 0.995 (99.5%) without YE.

Figures 14 and 15 display the contour and surface plots of total LA productivity for the same interactions, showing a similar pattern. LA productivity improved to over 0.6 g/L/h at sugar concentrations between 50 and 65 g/L and pH values from 7.90 to 8.90 (Figs. 14 and 15A). Additionally, LA productivity increased to more than 0.6 g/L/h at inoculum sizes ranging from 8 to 13% and pH values from 7.9 to 9.0 (Figs. 14 and 15B). Furthermore, LA productivity exceeded 0.6 g/L/h at a sugar level of about 80 g/L and pH values between 8.0 and 8.5 (Figs. 14 and 15C). LA productivity also increased to over 0.6 g/L/h when the temperature was 46 °C and inoculum sizes ranged from 8.0 to 13% (Figs. 14 and 15D). Moreover, LA productivity reached more than 0.6 g/L/h at a pH value of approximately 8.5, with inoculum sizes ranging from 9 to 13% (Figs. 15 and 16F) and temperatures between 43 and 53 °C (Figs. 14 and 15E).

### Optimization curves

**Fig. 17:**
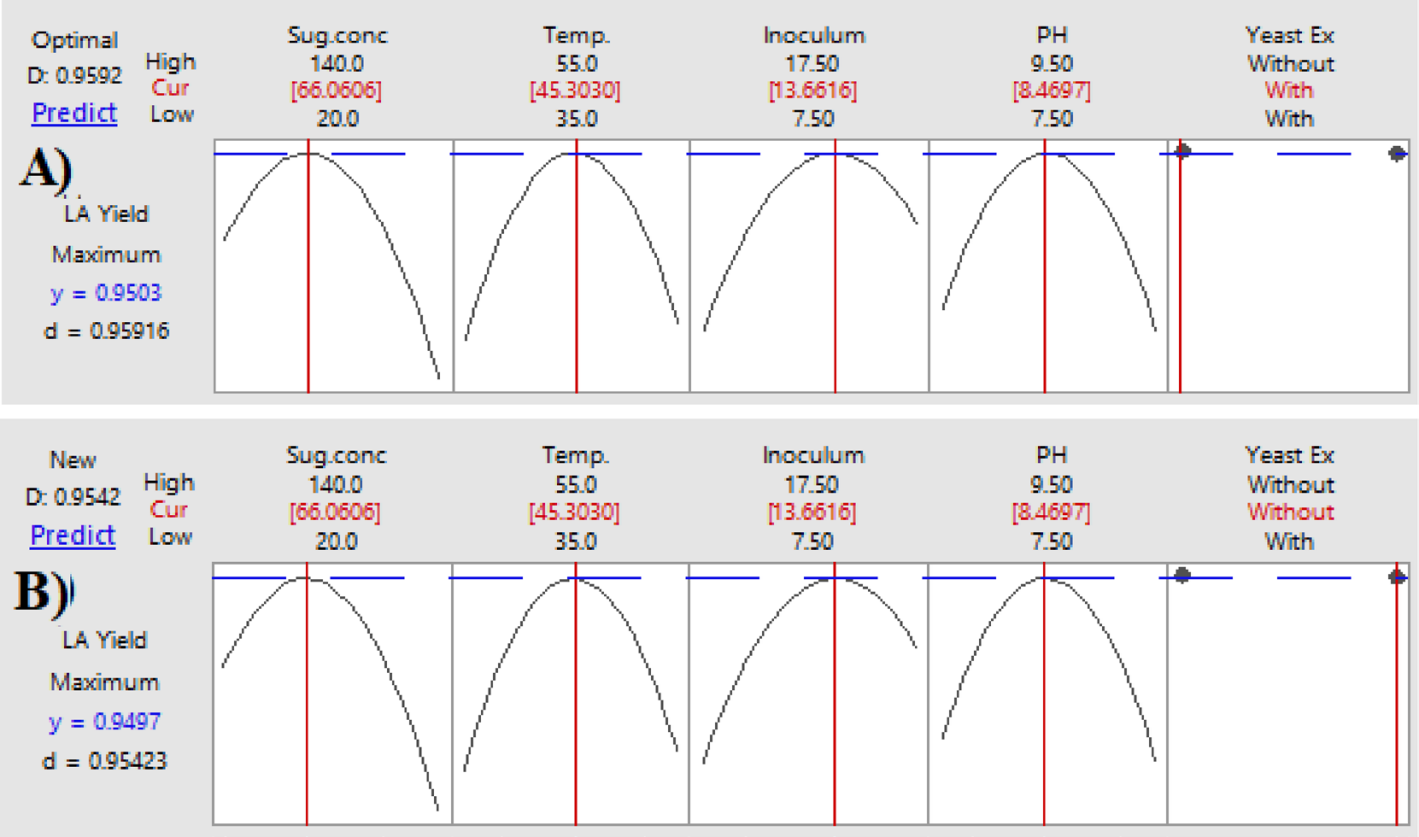
The factors’ effects on the anticipated responses (y) are displayed by the optimization curves, including maximum LA yield (g/g) with YE (**A**) and LA yield without YE (**B**) at low and high levels. The optimal factor settings (Cur) were predicted with a composite desirability (d) of 0.959 (95.9%) with YE and 0.954 (95.4%) without YE.

**Fig. 18:**
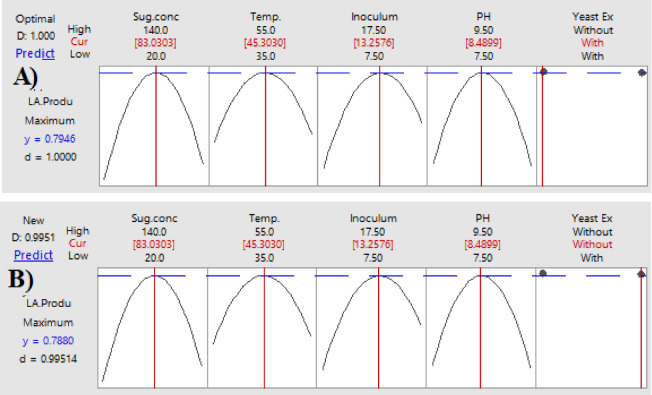
The factors’ effects on the anticipated responses (y) are displayed by the optimization curves including maximum LA productivity (g/L/h) with YE (**A**) and LA productivity without YE (**B**) at low and high levels. The optimum factor settings (Cur) were predicted with a composite desirability (d) of 1.00 (100%) with YE and 0.995 (99.5%) without YE.

**Fig. 19:**
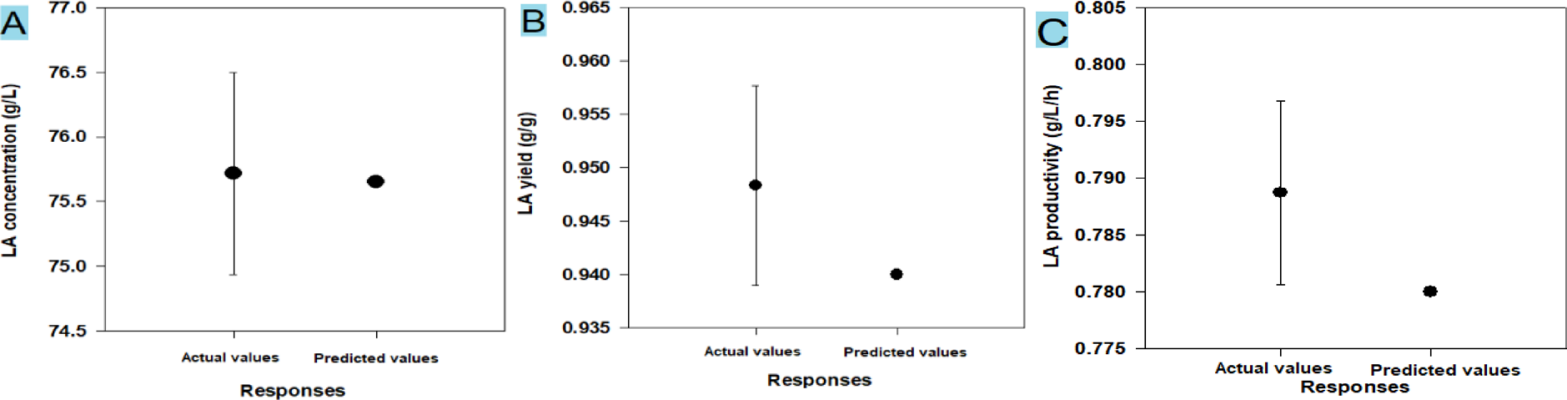
Scatter plot, comparing the actual (*n* = 10) and predicted (Pred.) values of LA concentration (**A**), LA yield (**B**), and LA productivity(**C**).

The optimization curves **(**Figs. 16 and 17, and 18**)** generated by the response optimizer tool display the final optimal values for variables that enhance LA concentration, yield, and productivity. Ten confirmatory runs were carried out under ideal circumstances to maximize LA production to 75.6 g/L (individual desire = 100%), increase LA yield to 0.94 g/g (individualـdesirability = 100%), and maximize LA productivity to 0.78 g/L/h. These conditions included an initial sugar concentration of 83 g/L, a temperature of 45.3 °C, a pH of 8.49, an inoculum size of 13.2%, and no YE supplementation (Fig. 19).

CSW contained several nitrogenous non-protein substances, including taurine, ornithine, ethanolamine, citrulline, and ɣ--aminobutyric acid. These compounds have biostimulant properties^19,84^ and serve as a sulfur source for aerobic microorganisms^85^, and are believed to be a primary energy source for many aerobic bacteria^86^. The growth of two *Rhodococcus* species using taurine as the sole nitrogen source was reported previously^87^.

To compare the actual values (mean ± SD) with the model-predicted values, the results of the confirmatory run were shown **(**Fig. 19**).** The real LA content was 75.7 ± 0.77 g/L, the yield was 0.94 g/L, and the productivity was 0.78 g/L/h, according to the data.

We successfully eliminated the need for additional nutrient supplementation in CSW, using it as the sole medium for growth. Although YE (YE) results in the highest LA concentrations due to its pyrimidine and purine bases and B vitamins^88,89^, its high cost—accounting for about 38% of total production costs—impacts the economics of LA fermentation^90^. Therefore, finding alternative, less expensive nitrogen sources is highly desirable. YE has been partially or entirely replaced by cheaper nitrogen sources, including those derived from agricultural waste and inorganic sources. However, only a few of these alternatives produced LA concentrations comparable to YE, even when combined with YE. Comparable LA levels in media need either longer fermentation durations or more expensive additives such as vitamins and peptone^91–94^. It is necessary to substitute more reasonably priced components for YE in the medium to sustain high output and yield. CSW, a byproduct of maize wet milling, may help address this issue. With its abundance of polypeptides, amino acids, and B-complex vitamins^19^, CSW is an excellent source of nitrogen for the majority of bacteria. We hypothesized that our initial study would maximize sugar utilization and LA production from CSW as a raw material, demonstrating the viability of CSW effluent as a cost-effective substrate for lactic acid biorefinery using strains like OP16-2, capable of converting sugars in CSW to LA based on a specific screening protocol.

### Pilot-Scale assessment (50 L bioreactor) for enhanced LA production from untreated CSW by *B. licheniformis* OP16-2

**Fig. 20:**
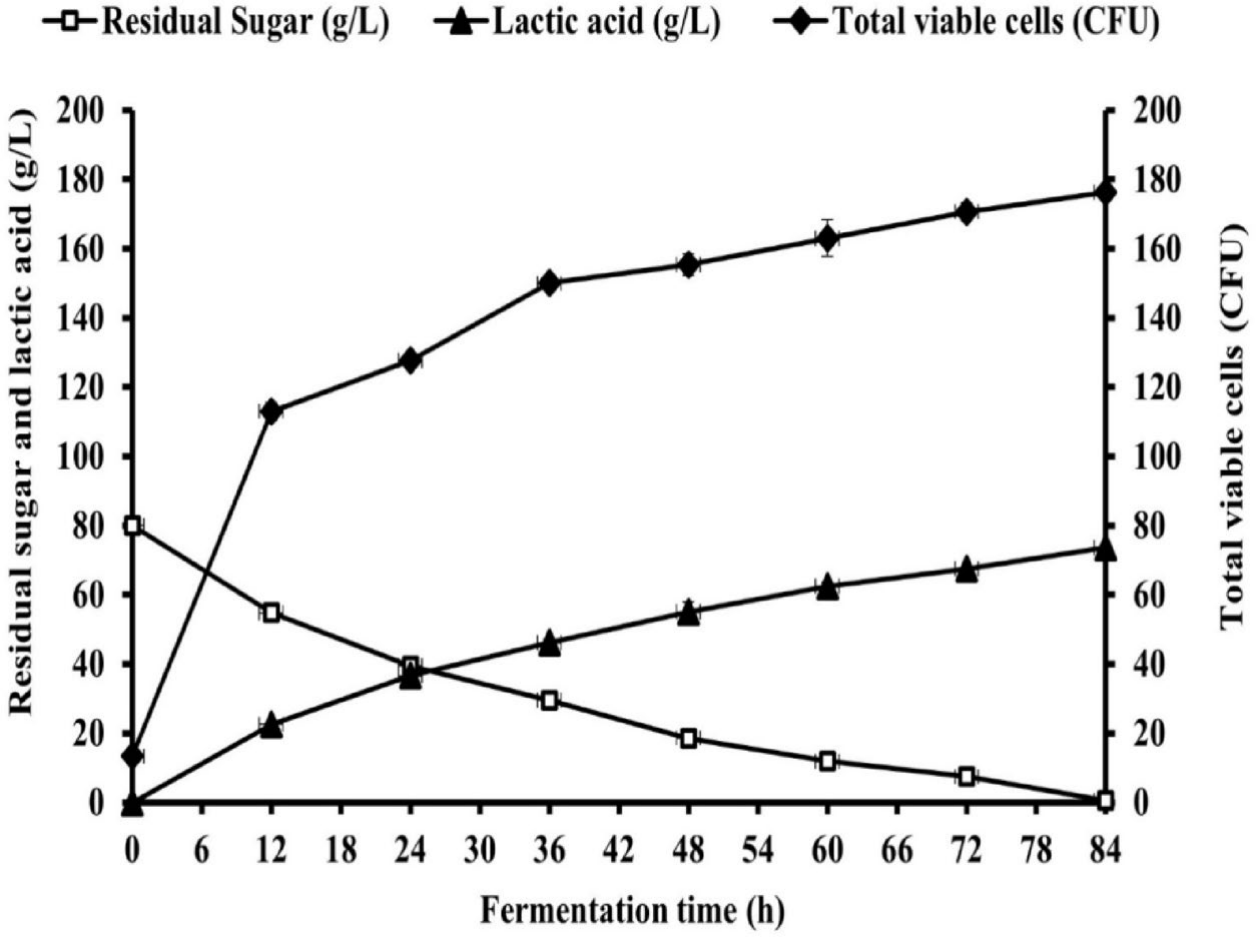
Batch fermentation for LA production by *B. licheniformis* OP16-2 in a bioreactor (50 L) that was maintained at 45 °C, and the pH was controlled at 8.49 ± 0.30 by 10 N NaOH for 84 h with an initial sugars of 80 g/L from untreated CSW. The standard deviation is less than the size of symbols if no error bars are seen.

The results in batch fermentation at Pilot-Scale (50 L bioreactor) for enhanced LA production from untreated CSW by *B. licheniformis* OP16-2 is shown in Fig. 20. It showed that the highest total viable count was increased as the fermentation time increased from 13.33 ± 2.08 × 10^10^ CFU/mL reaching the maximum values of 176.3 ± 1.52 × 10^10^ CFU/mL at the end of the fermentation process at 84 h (Fig. 20**)**. On the other hand, the sugars consumption pattern was shown to increase in the beginning of fermentation until the end of the fermentation process at 84 h when the sugars consumption ceased. The final LA concentration increased as the fermentation time progressed, reaching a maximum value of 73.6 ± 0.77 g/L at the end of the process. Interestingly, the LA yield ranged from 0.90 to 0.92 g/g of sugars consumed at all fermentation times. The maximum LA productivity was obtained at 12 h, with 1.88 ± 0.04 g/L/h, while total LA productivity was 0.87 g/L/h.

### Multi-pulse fed-batch LA fermentation with initial sugars of 80 g/L from untreated CSW in 50 L bioreactor

**Fig. 21:**
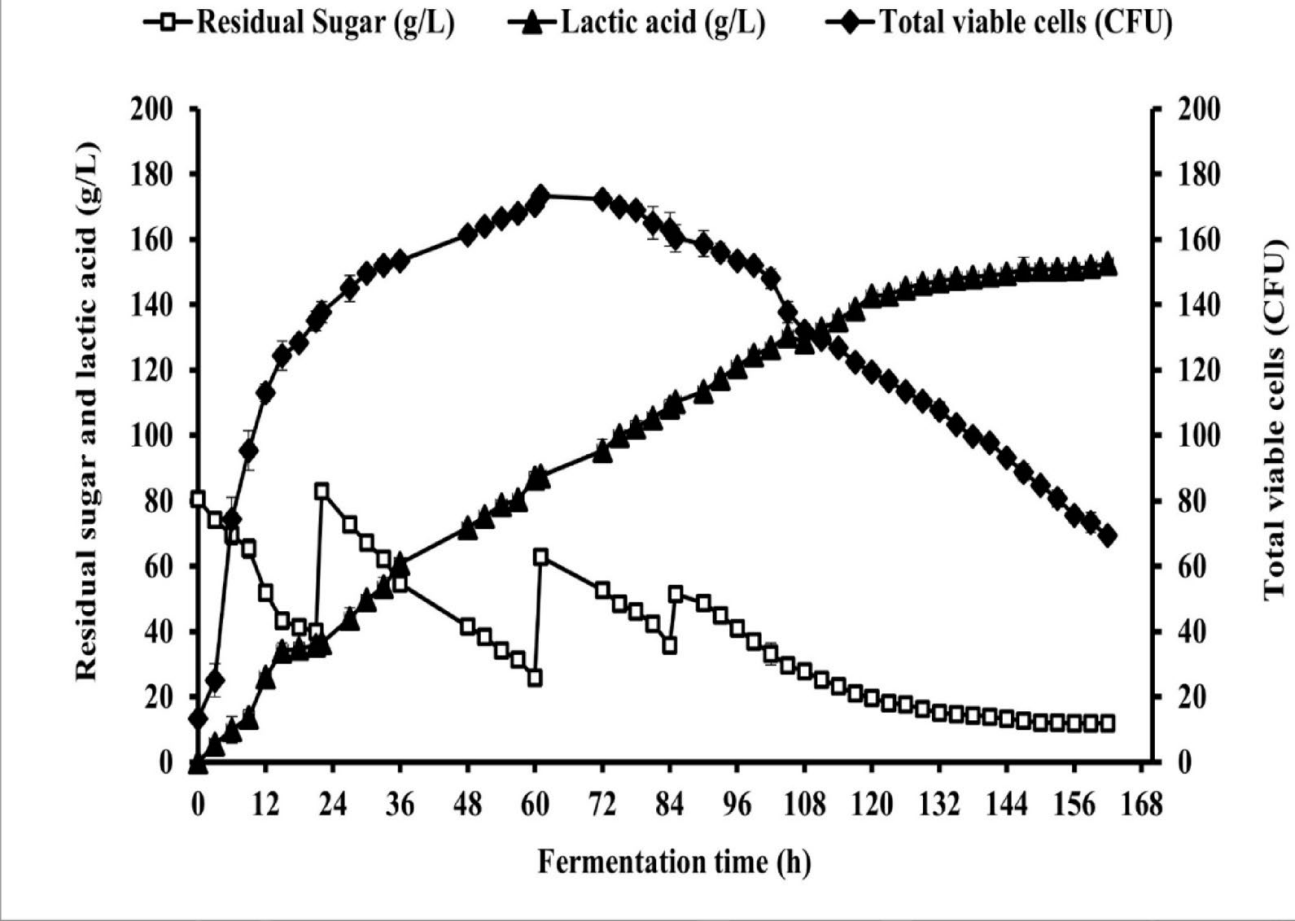
Multi-pulse fed-batch fermentation for LA production by *B. licheniformis* OP16-2 in a bioreactor (50 L) that maintained at 45 °C and the pH was controlled at 8.49 ± 0.30 with 10 N of NaOH for 162 h with initial sugars of 80 g/L from untreated CSW. The standard deviation is less than the size of symbols if no error bars are seen.

Pulse-feeding techniques produce significant levels of lactic acid with little effort^35,95–97^. The results obtained in Table 12, and represented graphically in Fig. 21, summarize the parameters for multi-pulse fed-batch LA fermentation by the OP16-2 strain. The pH was automatically controlled at 8.49 using 10 N NaOH. The fermentations were conducted at 45 °C in a bioreactor (50 L) containing 20 L working volume.

**Table 12:** Fermentation parameters for Pilot-Scale batch and Multi-pulse fed-batch fermentation for LA production by *B. licheniformis* OP16-2.

Fermentation mode	Total Viable Count (×10^10^)	Consumed sugar (g/L)	LA conc. (g/L)^a^	Y_LA_ (g/g)^b^	*P*_LA_ (g/L/h)^c^	Fermentation time (h)	Max *P*_LA_ (g/L/h)^d^ at the indicated time
Batch fermentation starting with 80 g/L of CSW	176.3 ± 1.5	79.3 ± 0.50	73.6 ± 1.70	0.92	0.87	84	1.88 ± 0.04(12)
Multi-pulse fed-batch fermentation starting with 80 g/L of CSW with several CSW feedings	69.3 ± 0.02	163.7 ± 0.55	152.6 ± 1.15	0.93	0.94	162	2.60 ± 0.12 (15)

During fermentation, multi-pulse feeding strategy were employed to add 40 g/L of concentrated CSW sugars when the residual sugars reached 35–40 g/L, and this process continued until the total added sugars reached 160 g/L. After that, 20 g/L of concentrated sugars were added until the total added sugars reached 180 g/L.

As shown in Fig. 21, the total viable count was increased as the fermentation time increased from 25 ± 5 × 10^10^ CFU/mL after 3 h, reaching the maximum values of 170.3 ± 1.52 × 10^10^ CFU/mL at 60 h, then stable after that to reach 170 ± 1.0 × 10^10^ CFU/mL at 75 h, while it gradually decreased after that to reach 137.6 ± 3.2 × 10^10^ CFU/mL at 105 h. Surprisingly, the total viable count continued to increase significantly after adding a second feed of 40 g/L of total sugars. In contrast, after adding 20 g/L of the concentrated sugars, the total viable count was rapidly decreased, reaching 69.3 ± 2.08 × 10^10^ CFU/mL at the end of the fermentation time at 162 h.

The sugar consumption pattern showed a gradual increase with increasing fermentation time, reaching a maximum value after 162 h., and then ceased. The maximum sugar consumption was 163.7 ± 0.55 after 162 h. Although sugar consumption showed a very slow increase, after adding the final feed of 20 g/L, especially at 105 h, an additional 11.8 ± 0.9 g/L of CSW sugars remained in the fermentation medium, which could not be utilized by the OP16-2 strain after 162 h of fermentation time. LA production exhibited an increase with the increase in fermentation time at varying titers. The production titer of LA was increases after all feedings of the concentrated sugars, but it was slowly increased after 105 h of fermentation time reaching a maximum LA concentration of 152.6 ± 1.15 g/L after 162 h.

It’s interesting to note that the LA yield gives comparable values at all fermentation times, ranging from 0.86 to 0.93 g/g of sugars consumed. The total LA productivity was 0.94 g/L/h after 162 h, while. the maximal LA productivity was obtained after 15 h of fermentation with 2.6 g/L/h.

Comparison between the batch mode and fed-batch mode indicates that the latter produced higher levels of lactic acid concentration, yield, and productivity^98^. By maintaining a low substrate concentration during fermentation and minimizing the inhibitory effects of sugar and osmotic pressure on bacterial cells, the fed-batch mode can increase the final product content in the bioreactor^99^. The fed-batch mode outperformed the batch mode in terms of performance because it was able to reduce the high viscosity caused by the large biomass load and prevent the medium’s mass and heat transfer efficiency from being inhibited^100^. Another justification was that by feeding the medium with the substrate at various periods, the inhibition of the product and substrate might be avoided^101^.

## Conclusion

This study developed a cost-effective method for enhancing LA production from CSW under thermo-alkaline conditions, thereby reducing contamination risks. *B. licheniformis* OP16-2 showed strong resistance to various inhibitory compounds, making it a suitable candidate for LA production from CSW effluent without any nutritional supplementation or treatment processes. Optimizing batch fermentation conditions using the OFAT method increased LA production, achieving a maximum concentration of 73.6 ± 1.73 g/L, a yield of 0.94 g/g-consumed sugar, and a productivity of 0.87 ± 0.02 g/L/h with 80 g/L CSW sugar, an inoculum size of 12.5% (*v/v*), at 45 °C and pH 9.0 using NaOH as a neutralizing agent. Statistical optimizations revealed that LA production under optimized fermentation conditions was higher than that of classical methods, with 75.7 ± 0.77 g/L, 0.94 g/g, and 0.78 g/L/h for LA production, LA yield, and LA productivity, respectively. We also succeeded in eliminating the need for additional nutrient supplementation in CSW, using it as the sole medium for growth. Furthermore, Pilot-Scale multi-pulse-fed batch fermentation was assist to produce LA in a long-term, economical strategy. This increased lactic acid concentration to 152.6 ± 1.15 g/L with a high yield (0.93 g/g) and total LA productivity of 0.94 g/L/h in a 50 L bioreactor maintained at 45 °C and pH controlled at 8.49 ± 0.30 using 10 N NaOH for 162 h.

## Data Availability

The data used to support the findings of this study are available from the corresponding author upon request. **Bacillus licheniformis** was isolated from soil samples, identified, and then deposited in NCBI GenBank with gene accession number ON650717 **https://www.ncbi.nlm.nih.gov/nuccore/ON650717.1**.
